# Model-based Comparisons of the Abundance Dynamics of Bacterial Communities in Two Lakes

**DOI:** 10.1038/s41598-020-58769-y

**Published:** 2020-02-12

**Authors:** Phuongan Dam, Luis M. Rodriguez-R, Chengwei Luo, Janet Hatt, Despina Tsementzi, Konstantinos T. Konstantinidis, Eberhard O. Voit

**Affiliations:** 1 Department of Biomedical Engineering, Georgia Tech, Atlanta, GA USA; 2 School of Civil and Environmental Engineering, Georgia Tech, Atlanta, GA USA

**Keywords:** Water microbiology, Limnology

## Abstract

Lake Lanier (Georgia, USA) is home to more than 11,000 microbial Operational Taxonomic Units (OTUs), many of which exhibit clear annual abundance patterns. To assess the dynamics of this microbial community, we collected time series data of 16S and 18S rRNA gene sequences, recovered from 29 planktonic shotgun metagenomic datasets. Based on these data, we constructed a dynamic mathematical model of bacterial interactions in the lake and used it to analyze changes in the abundances of OTUs. The model accounts for interactions among 14 sub-communities (SCs), which are composed of OTUs blooming at the same time of the year, and three environmental factors. It captures the seasonal variations in abundances of the SCs quite well. Simulation results suggest that changes in water temperature affect the various SCs differentially and that the timing of perturbations is critical. We compared the model results with published results from Lake Mendota (Wisconsin, USA). These comparative analyses between lakes in two very different geographical locations revealed substantially more cooperation and less competition among species in the warmer Lake Lanier than in Lake Mendota.

## Introduction

The health of our lakes is of utmost importance, especially if they serve directly or indirectly as reservoirs for our water and food supply. A concern is that many of these lakes are also used for recreational activities and are surrounded by agriculture, which exposes them to pollution from motor boats and run-off. The water quality of lakes is heavily dependent on the interactions among thousands of bacterial species, archaea, phyto- and zoo-plankton, protists and viruses. The sheer numbers of species in these complex communities render health assessments of lakes difficult. For instance, diverse communities, with many interactions among community members, are generally considered good markers of healthy ecosystems, as long as pathogens or invasive species are absent. Indeed, diversity within microbial communities has been associated with the robustness and the resiliency of ecosystems^1^. It is challenging to assess species-species (or OTU-OTU) interactions, because they occur and change dynamically in environments that undergo substantial seasonal fluctuations. They are also “asymmetric” in a sense that the effect of OTU A on OTU B is not necessarily the same as the effect of B on A. Confounding this challenge is the overwhelming number of participating species, many of which are difficult—if not impossible—to culture in the laboratory^2^.

Here we proffer that the prudent choice of a computational modeling framework and of customized methods of analysis permit insights into the dynamics of complex microbial lake communities that are not achievable with traditional correlation network models. The choice of a mathematical modeling framework is not trivial, as there are very few nature-given guidelines; we simply do not know what types of models are “true”^3^. Ultimately, any model choice is a compromise between biological relevance, the repertoire of system behaviors a model can capture, model fits, and analytical tractability. In the past, graph-based network models^4–6^, multivariate autoregressive models^7–9^ and dynamic systems models^10–12^ have been prevalent approaches for the assessment of interactions among populations; key features of these approaches are described in the *Discussion*. Our analysis here confirms an earlier study^13^ indicating that, among the available modeling choices, dynamic Lotka-Volterra (LV) models provide a good compromise. In particular, we demonstrate that the LV approach allows minimally biased comparisons between the features of different communities and their responses to perturbations. In the long term, the scientific community should engage in many such comparisons of (seemingly) similar or (evidently) different ecological systems in order to understand the general principles governing the dynamics of communities in these systems, because such an understanding is a prerequisite for targeted, effective interventions and sustainable water management.

We illustrate the value of model-assisted comparisons by contrasting our primary results for Lake Lanier (State of Georgia, Southeast USA) with corresponding results from the rather different Lake Mendota (State of Wisconsin, Midwest USA). Lake Lanier is a large water reservoir that provides drinking water for about 5 Million people. It contains in its surficial photic zone over 11,000 microbial Operational Taxonomic Units (OTUs). These were defined at the 97% 16S/18S rRNA gene nucleotide identity level (16S or 18S for simplicity hereafter), and over 97% of them are bacterial. The OTUs show distinct patterns in abundance throughout the year^14^. Slightly more than 1,400 OTUs (13%) constitute 87.2% ± 2.5% of the total microbial cell count, depending on the time of the year, while the remaining OTUs are considered rare^15^. We measured the OTU abundances recurrently between 2010 and 2015, which resulted in a large dataset containing measurements at 29 time points that capture the bacterial community dynamics of the abundant species in Lake Lanier. All samples represent a water depth of 5 meters and were taken at the exact same site (Browns Bridge)^14^. Maybe not surprising, the OTU composition of the complex community changes dramatically throughout the year, and the dynamics of these changes is an important determinant of the functionality of the lake.

Previously, we analyzed the bacterial community of Lake Mendota, Wisconsin^13,16,17^, for which we employed different variations of a slightly modified Lotka-Volterra (LV) model that dramatically simplified the parameter estimation to standard linear regression^18^. The models were used in two variants. The first accounted for the interactions among 14 sub-communities (SCs), which we defined pragmatically through time-dependent clustering of OTUs, based on their blooming during the same time periods of the year, and three environmental factors (ENVs). The second variant addressed the interactions between individual OTUs on the one hand and the SCs and ENVs on the other. Adapting modeling strategies similar to those applied to the Lake Mendota data, we present here modeling results for Lake Lanier, including the effects of pairwise interactions between bacterial sub-communities and the effects of environmental factors on the seasonal abundances of each sub-community. Archaea and higher organisms were not explicitly considered but their impact is to some degree implicitly captured by the LV parameters. The proposed model provides a flexible framework for integrating available data into a single computational structure, identifying important environmental factors, characterizing dynamically changing, asymmetric, bi-directional species-species interactions of interest, and performing thought experiments regarding the effects of slight perturbations in the conditions affecting the lake. Furthermore, the comparison of results between lakes offers novel insights into the relationships between bacterial communities and environmental factors, as well as the prevalence of cooperation or competition among bacterial species.

## Results

### Physical and chemical characteristics of two lakes

The physical and chemical measurements of Lake Lanier (GA) and Lake Mendota (WI)^16,17^ were superimposed into one ‘representative’ year for each lake (Fig. S1). We compared six physico-chemical markers that were measured in both lakes, namely water temperature, ammonia, nitrite + nitrate, phosphorus, organic carbon, and pH, and found that they differ considerably (Table S1, Fig. S1). The water temperature in Lake Mendota is generally 4–5 °C lower than in Lake Lanier, with much greater differences in April (6.2 °C versus 17.2 °C). The ammonia (0.50 ± 0.1 versus 0.17 ± 0.12 mg/L) and nitrite + nitrate (0.67 ± 0.25 versus 0.38 ± 0.09 mg/L) concentrations are about twice as high at peak level in Lake Mendota. The total phosphorus and organic carbon concentrations of Lake Mendota are also higher, as is its pH (8.5 ± 0.2 versus 7.3 ± 0.4). Further details can be found in the *Supplements*.

### Year-around abundance patterns of 14 sub-communities

**Figure 1:**
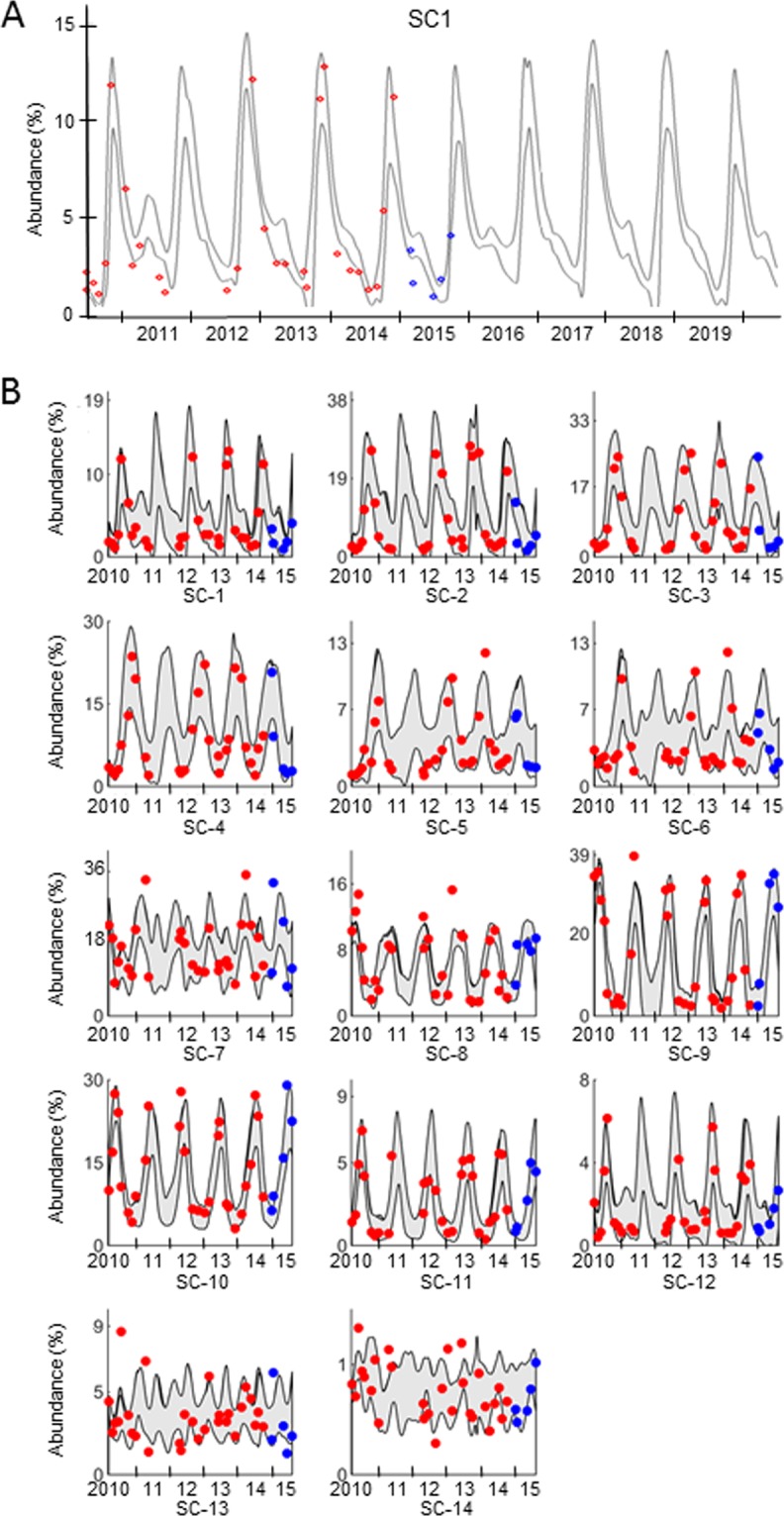
Observed, modeled, and predicted annual abundances of 14 bacterial sub-communities in Lake Lanier. The abscissa shows the days of a multi-year period between July 2010 and October 2015, and the ordinate exhibits the relative abundances (percentages) of bacterial sub-communities; note the different scales of the ordinates. Measurements, indicating time points and abundances, are shown as red dots. They were used to estimate the parameters for the model. The ranges (mean ± 2 standard deviations) predicted by our 84 best-performing model parameterizations (*m* = 3) for this time period are shown in grey. Data collected between March and October of 2015 (blue) were used as validation. It would have been desirable to have much denser and longer time series data for validation, but we chose this short period as a balance between model training and validation. Panel A: Ten-year model results and validation with data not used before (blue dots) for SC-1. Panel B: Model fits (red dots) and validation with data collected between March and October of 2015 (blue) for all sub-communities. Predictions of trends were computed for five more years, but are not shown. Note that SC-13 and SC-14, which contain OTUs not fitting into other SCs, are much less defined than the other SCs.

The abundances of most OTUs in Lake Lanier clearly follow seasonal patterns (Fig. S2). Clustering, as described elsewhere^13^, reveals that twelve of the 14 sub-communities in the lake peak once per year, while SC-13 and SC-14 peak twice (Figs. 1 and S3). Superposition of six years into one representative year (Fig. S3) demonstrates that the annual profiles of each sub-community are very similar across sampling years. It also reveals that the peaks of the first twelve SCs are significant, with abundances that are 2.7 to 11.6 times higher at the peak than for the low-level abundances during the rest of the year. Throughout the year, the total abundances of SC-1 to SC-12 collectively constitute between 90.5 and 98.0% of the entire bacterial community.

The classification of bacterial species into SCs, based on blooming periods, enabled direct pairwise comparisons between corresponding SCs in two lakes. In both lakes, for example, SC-1 is comprised of bacteria belonging to similar taxonomical groups that are highly abundant in January. Comparisons of the 14 SCs reported in Lake Mendota and Lake Lanier reveal that their annual abundance profiles are correlated, although their peak heights are quite different (Fig. S4). In particular, a comparison of the abundances of corresponding SCs at their peak times shows that nine out of the twelve SCs with one annual peak have higher abundances in Lake Mendota compared to their counterparts in Lake Lanier. Among these, the three SCs with the largest differences (more than two-fold) are SC-1, SC-12 and SC-5. Generally, our observations suggest that bacteria in the colder Lake Mendota exhibit more distinct annual patterns compared to those in the warmer Lake Lanier, which may not be surprising.

### Model results characterizing the dynamics of sc abundances

#### Trends in sub-communities

We used the Lotka-Volterra modeling format (see *Methods*) to characterize the relationships between the abundances (*X*_*i*_, *X*_*j*_) of pairs of species, OTUs, or SCs *i* and *j*. Parameters for the pairwise interactions between *X*_*i*_ and *X*_*j*_ are termed *α*_*ij*_, while interactions between *X*_*i*_ and environmental factors *T*_*k*_ are called *β*_*ik*_. We included in the model three environmental factors, which we had identified as critical (see following section for details).

Overall, the simple LV model matches the data from the complex lake environment surprisingly well, and the trends in dynamic changes of OTU abundances are consistently preserved (Fig. 1A). Specifically, 78% of the observed data are within the mean ± 2 standard deviations of the predicted values during the time period 2010–2014, which we used for parameter estimation. Similarly, 71% of the observed data between March and October of year 2015, which were not used in the model construction, are within the mean ± 2 standard deviations of the predicted values.

To test whether future trends in abundances could be predicted, we used the data collected from 2010 to 2014 and estimated the parameters for the model. We then used the model to predict the abundances of the 14 SCs for the next 10 years, starting with the initial values from the first observed dataset of July 2010. The predicted abundances for 2015 were compared to abundances actually observed between March and October of 2015, indicating good consistency (Fig. 1A,B). Of course, caution is necessary with all predictions into the far future. For example, any extreme black-swan events or extreme perturbations are not reliably covered by the LV model or any other model, such as an autoregressive model^9^.

#### Effects of environmental conditions

To investigate which combination of environmental factors significantly affects the abundances of the 14 identified SCs, we made abundance predictions with a large number of parameterizations of the Lotka-Volterra model, by investigating all single factors and all combinations of up to 8 (*m* = 2,…, 8) among the 12 physical and chemical factors measured. This number was sufficient, due to strong correlations among several of the environmental factors. The estimated parameters were represented in the *β*_*ik*_ terms of the model.

The inclusion of water temperature significantly improved the abundance predictions, and addition of pH and the sulfate concentration improved the predictions further, whereas including other environmental factors did not significantly improve predictions, when the number of parameters was taken into consideration^19^. The model with these three environmental factors will be referred to as “*m* = 3” model, whereas the model accounting only for temperature is termed the “*m* = 1” model (for details, see *Supplements*). As a side note, we focused on temperature, pH and sulfate, but any condition directly correlated with these could be the true drivers of the system.

To assess uncertainties and their effects on parameterizations, we determined *ensembles* of differently parameterized models leading to similar fits. Specifically, we randomly selected 200×*p* parameterizations, where *p* is the number of parameters being estimated (for details, see *Supplements*). In each case, we accounted for up to three of the measured environmental factors. For each estimation, we slightly varied the upper bound for the key parameters -*α*_*ii*_, which represent crowding or competition within the *i*^th^ SC, as described in the *Methods* section. Among the thousands of results, ensembles of 193 (for *m* = 1) or 84 (for *m* = 3) parameter sets, respectively, reflected the observed data with a similar, small residual error. All model parameterizations within these ensembles capture the observed dynamic trends in SCs abundances remarkably well (see results in Fig. 1 for *m* = 3; results for *m* = 1 not shown).

**Figure 2:**
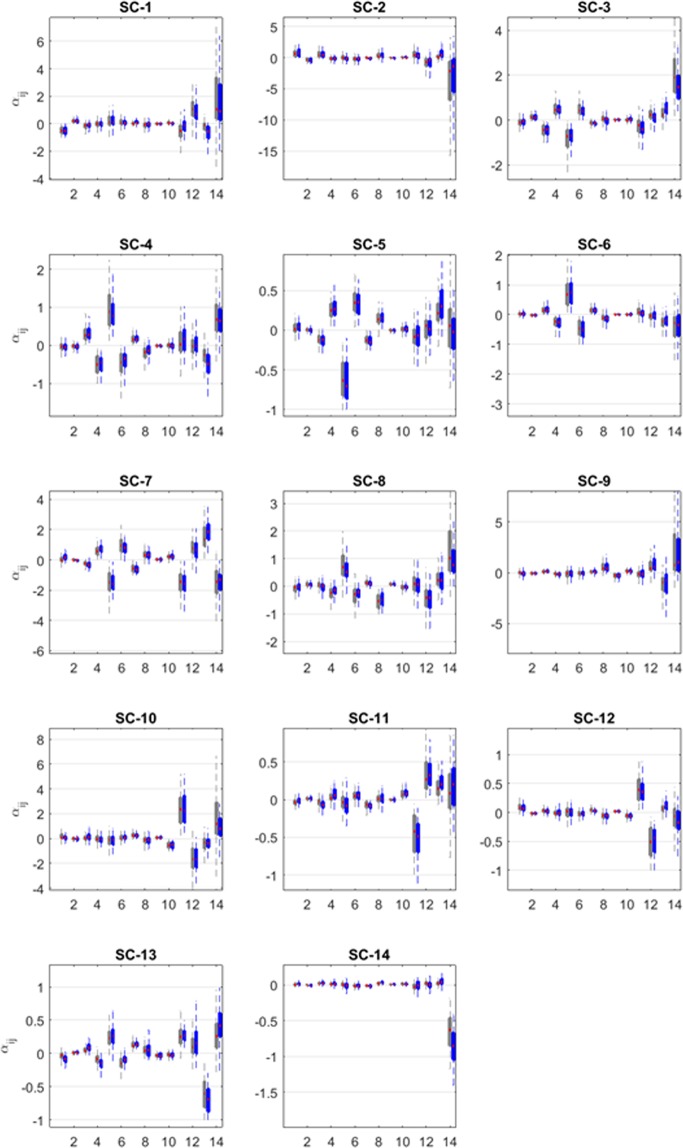
Estimated values of interaction parameters (*α*_*ij*_) between pairs of the 14 sub-communities in Lake Lanier, when one or three environmental factors are taken into account. The plot shows *α*_*ij*_ values of SC-1 to SC-14. In each subplot, the median (red), the first and third quantiles (heights of boxes) and the range of 99.3% of the values (lengths of the whiskers) are shown. For each *α*_*ij*_, the pairs of boxplots correspond to one (left; grey) or three (right; blue) environmental factors. Between 46% and 60% of all *α*_*ij*_’s are negative, indicating competition.

**Figure 3:**
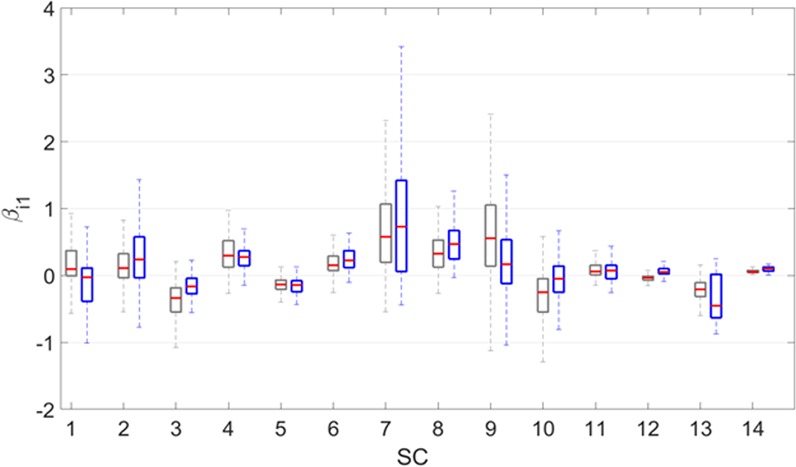
Estimated parameter values associated with temperature (*β*_*i*1_) and the 14 sub-communities. The plot shows *β*_*i*1_ values for SC-1 to SC-14. The pairs of boxplots correspond to *m* = 1 (grey) or *m* = 3 (blue) environmental factors of 193 (*m* = 1) or 84 (*m* = 3) models. Specifically, the median (red), the first and third quantiles (heights of boxes), and the ranges of 99.3% of the values (lengths of the whiskers) are shown. Most of the 14 *β*_*i*1_ values have similar medians in the two models, whereas *β*_*i*1_ for SC-1 and SC-12 are somewhat different. However, the latter two *β*_*i*1_ values are relatively small.

Comparing the dynamic models that only include water temperature (*m* = 1) or all three environmental factors (*m* = 3) demonstrates that the two models have different parameterizations, but that the positive or negative trends in the interaction parameters *α*_*ij*_ are quite consistent. Moreover, the means and standard deviations of all *α*_*ij*_ values do not exhibit significant differences between the two models in 85.7% of all *α*_*ij*_ pairs (including *α*_*ii*_) (Fig. 2). In stark contrast, and presumably not surprising, 50% of all *β*_*i*1_ values, which are the parameters representing the effect of water temperature on the *i*^th^ SC, are significantly different between the two models (Fig. 3). Nonetheless, most of the positive or negative signs among *β*_*ik*_ values do not change within each ensemble of solutions for *m* = 1 or 3. Details are presented in the *Supplements*. Interestingly, autoregressive models also tend to characterize signs much better than magnitudes^9^.

**Figure 4:**
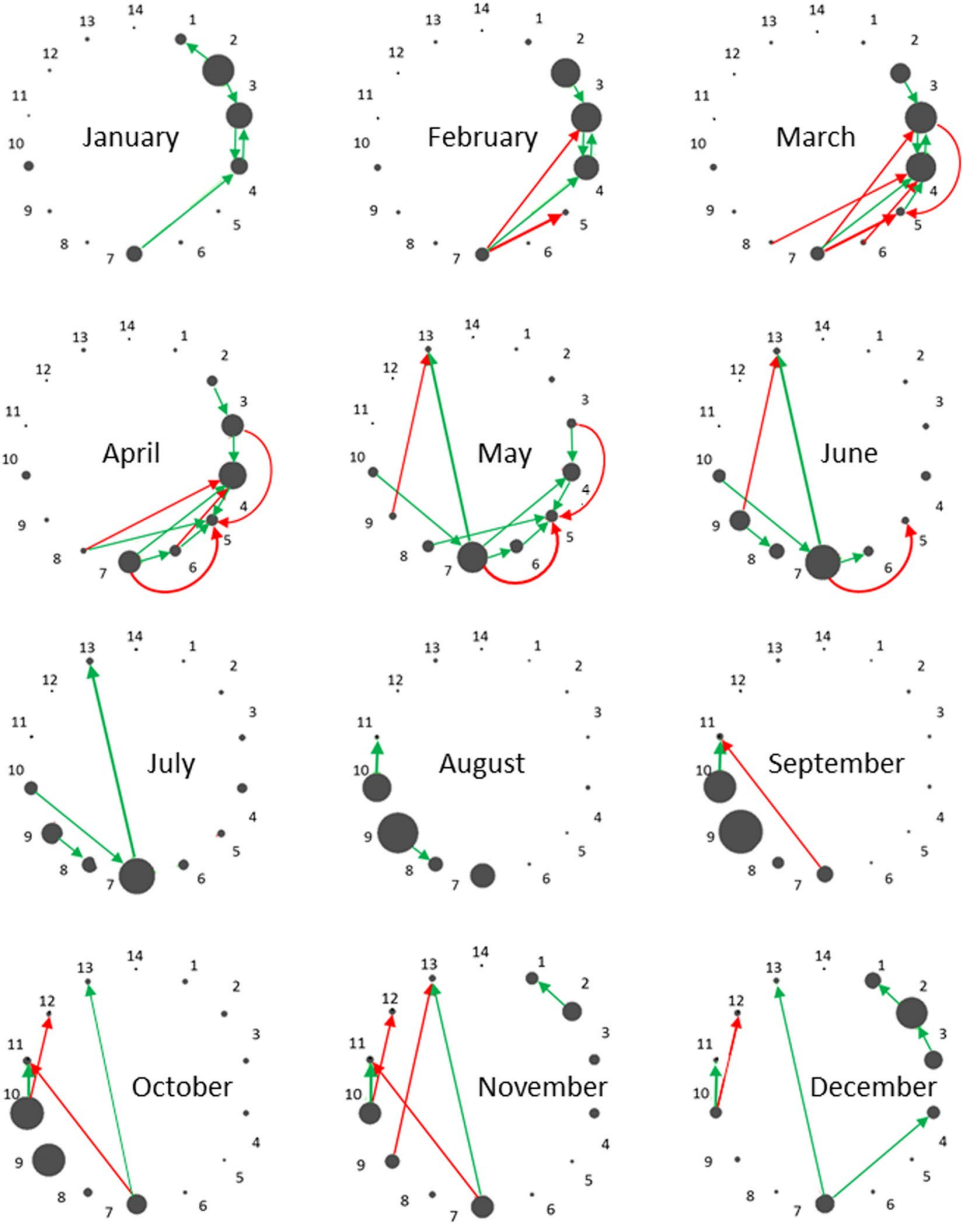
Networks of strongest interactions among the 14 sub-communities by month. The interaction network for each month was computed from the best sub-community model and weighted by the monthly average abundance of the sub-communities. The means and standard deviations of all values were computed, and only those interactions were retained that are at least two standard deviations away from the mean. This cut-off corresponds to 5% of all interactions. Each vertex size is proportional to the size of the sub-community.

The estimated interaction values *α*_*ij*_ allowed us to describe the dynamic interactions of 14 SCs in Lake Lanier throughout the year (Fig. 4). These results suggest that the temporally changing interaction network is driven by highly abundant SCs whose blooming periods are close to each other. The interaction parameters were also used to compare the models between the two lakes.

### Predictions of the effects of moderate environmental perturbations

The LV model allows predictions that are based on the full system dynamics, and not merely on correlation coefficients, because the underlying time series data are not independent but reflect continuous trends. Ideally, the LV model and its best parameterizations are close to the truth in capturing interactions and the effects of environmental factors. Of course, we do not know that. However, if we trust the model, we can cautiously make predictions of the future trends in the bacterial lake community under moderately changed environmental conditions, which is not possible with simple static models. Such predictions assume that perturbations pertain to factors represented in the model and are moderate, and that no unexplainable black-swan events occur.

**Figure 5:**
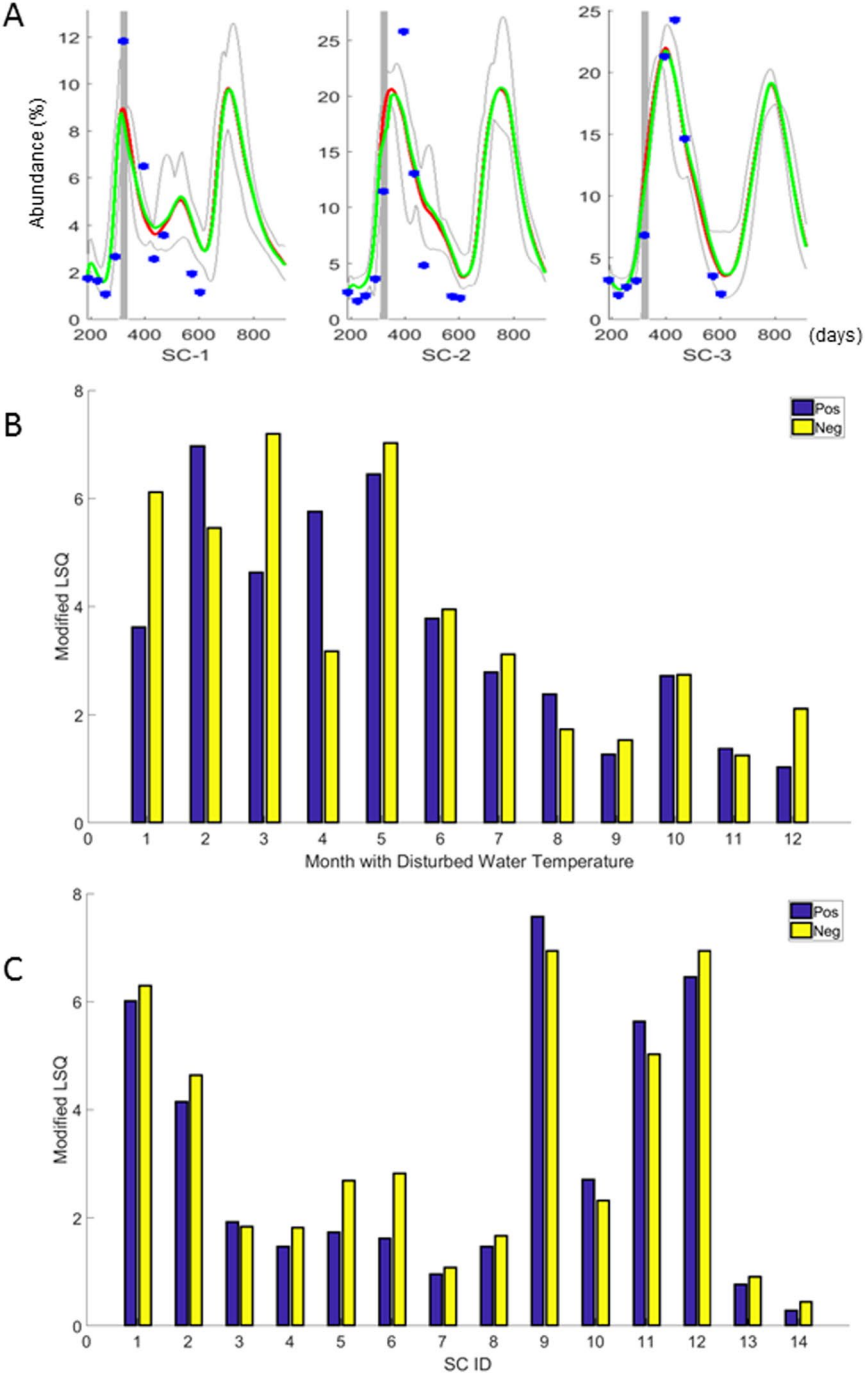
Predicted changes in abundances in response to altered temperature. For each environmental factor, the maximum observed variation was used to modify the measured environmental factor over a 30-day period. The change was initiated at the beginning of each calendar month. Panel A: Examples of predicted abundance changes (red/green lines) over time for each SC were simulated with either the original water temperature (red) or a water temperature (green) that was modified during a 30-day period (grey bar). Observed values are shown as blue dots. The abscissa shows the days of a two-year period starting from 7/1/2010. The ordinate shows the relative abundances (percentages) of SC-1, SC-2 and SC-3. The maximum and minimum (grey) of the predicted values are shown. Panel B: Results were grouped by the month during which water temperature was modified. The water temperature was either increased (blue bars) or decreased (yellow bars) for 30 days corresponding to calendar months, and a modified LSQ was computed using the mean abundance of 10 simulations using 10 randomly chosen sets from among 84 estimated models. The results suggest that a 30-day change in water temperature between January and May has a stronger effect on SC abundances. This result was validated with observed data (*p* = 0.03). Panel C: Results were grouped by SCs. For each of the 14 SCs (abscissa), the modified LSQ was computed when the water temperature was either increased (blue bars) or decreased (yellow bars) for 30 days. The change in water temperature has a greater effect on some SCs (1, 2, 9, 11, 12) than on others. This observation was confirmed with observed data (*p* = 0.0046).

To explore future trends, quasi through thought experiments, we randomly chose 10 parameter sets from the ensemble of 84 well-fitting model parameterizations, which each account for 14 SCs and three environmental conditions (*m* = 3). We used these 10 parameterizations to predict the abundances of all SCs when environmental conditions were changed from the average patterns, as described in the *Methods* section. In order to alter the environmental conditions in a realistic manner, we first analyzed their natural variability. Specifically, we computed the largest observed difference from the average pattern. For simulations of perturbations, we increased or decreased the value of any one specific environmental condition by this observed difference for a period of 30 days. In other words, each environmental condition was changed to the observed high or low for 30 days. An example, shown in Fig. 5A, suggests that the lake system tolerates such changes very well. The resulting abundance values for each SC demonstrate that changes in water temperature have stronger and longer lasting effects than changes in pH and sulfate (Supplement Table S2). Specifically, changing the water temperature, pH, or sulfate as described resulted in average maximal changes in SC abundance values of 13.7% ± 6.5%, 6.0% ± 5.0% and 1.7% ± 2.5% for water temperature, pH and sulfate, respectively.

Further analysis demonstrated that the effect of a one-month rise or decrease in water temperature on the abundance of the SCs is differently strong throughout the year. Specifically, the simulation results suggest that a change in water temperature causes a larger change in the predicted abundances of SCs if it happens at some time between January and May rather than between June and December (Fig. 5B). It is obviously not possible to validate this prediction directly, as it pertains to an actual lake, where the temperature must not be altered artificially (see *Supplements* for an indirect validation). In addition, the simulation results suggest that a change in water temperature during the cooler months of the year has a stronger effect on the SCs’ blooming periods than a change in warmer months (except for SC-10) (Fig. 5C). To test the significance of this result, we categorized the SCs by their blooming time into two groups, consisting of SCs 1, 2, 9, 11 and 12 in the first group (fall and winter) and the other SCs in the second group (spring and summer). Analysis of variance revealed that the average change in observed abundances of SCs per unit of change in water temperature is significantly different between the two groups of SCs (*p* = 0.0046). This result may not be surprising, but it is comforting that the model, quasi automatically (*i*.*e*., without being trained to do so), reflects the biological expectation, rather than making unreasonable predictions. As is well known, no model is ever completely true, but our model “survives” this particular validation test.

With regard to the pH level, the simulation results suggest that the effect of a disturbance is stronger if it happens in January or February, or between August and October. These time periods coincide with particularly low or high levels of pH. While the model predictions were quite clear, we were unable to prove significance (*p* = 0.2) with the strategy we used for temperature. Similarly, the simulation results suggest that the effect of disturbances in sulfate is stronger in January and February. The reasons for lacking significance are unclear, but it is notable that the patterns in the pH and sulfate concentration data are not as crisp as the temperature data.

### Pairwise interactions among the 14 sub-communities in lakes lanier and mendota

Lakes Lanier and Mendota differ considerably in their geographical locations, their annual temperature profiles, and presumably many other physicochemical characteristics (see *Supplements*). It is therefore interesting to compare key findings from this analysis with our earlier results from Lake Mendota^13^.

Analyzing all successful parameterizations of the SC model for Lake Lanier, with account for either one (*m* = 1) or three (*m* = 3) environmental factors, yields ensembles of 193 (or 84) satisfactory solutions, respectively. Using these parameterizations, we computed pertinent statistics regarding their parameter values (Fig. 2). Interestingly, the estimated *α*_*ij*_ and *β*_*ik*_ values, representing OTU interactions and the effects of environmental factors, respectively, are in most cases quite consistent. Between 46% and 60% of all *α*_*ij*_’s are negative (*m* = 1, 3), indicating competition, while others are positive, suggesting cooperation between SCs. The results for Lake Mendota indicate a noticeable higher level of competition (62.0–67.0%).

The *α*_*ij*_ matrix is asymmetrical, because interaction effects are typically not reciprocal, *i*.*e*., the effect of OTU A on OTU B may differ from that of B on A. Among the pairs (*α*_*ij*_, *α*_*ji*_) from Lake Lanier, roughly 40%, 10%, and 50% are −/−, +/− or +/+, respectively. In stark contrast, the corresponding percentages for Lake Mendota are about 75%, 5% and 20%, respectively, which again points to much higher competition in the colder lake. It may be tempting—but is difficult—to interpret the direct particular biological relevance of the *α*_*ij*_ values as we do not yet have genome sequence information for validation and >95% of the OTUs in total represent novel, not-yet described species, which lack information on encoded metabolic pathways and requirements for growth. Nonetheless, we describe an example of our results below as an indication of what these interaction terms may mean (see *Supplements* for further details).

The analysis of pairs of *X*_*i*_ and *X*_*j*_ identified a cluster containing three OTUs in two taxonomical groups, including the order *Sporichthyaceae* (EU800077/AbmC1553 and AY752123/Bctrm577) and the family *Acidimicrobiaceae* (FJ827858/M21eMend). *Sporichthyaceae* have been described as facultative anaerobes^20^, while *Acidimicrobiaceae* are obligate acidophiles that can oxidize ferrous (Fe^+2^) or reduce ferric ion^21^. Our dynamic models allowed us to predict the abundance of FJ827858 from the abundance of AY752123. In contrast, the abundances of the other two OTUs cannot be predicted by their cluster partners. This result suggests that AY752123 is likely to affect the abundance of FJ827858 while the opposite is not true. Because FJ827858 belongs to the obligate acidophiles that use iron metabolism, one should explore whether AY752123 does so once its genome sequence becomes available. Similar (potential) interactions were observed between families that are primary producers (*Cyanobacteria*) and organic heterotrophs (various *Proteobacteria*) (data not shown but are available on our websites).

**Figure 6:**
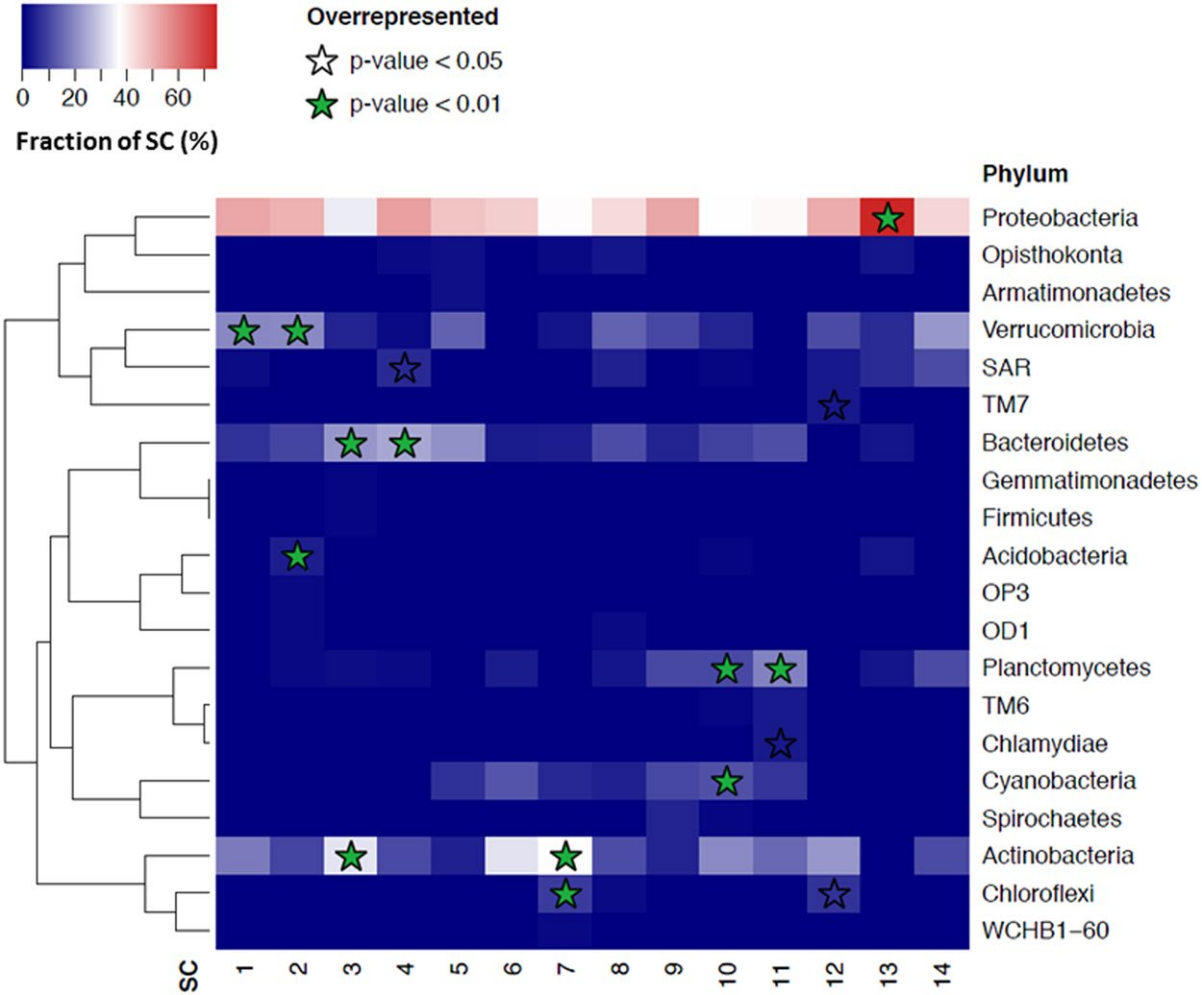
Identification of overrepresented phyla in different sub-communities of Lake Lanier. Results of Fisher’s exact test analysis on 2 × 2 contingency tables of target phylum *versus* other phyla, and target SC *versus* other SCs, indicating overrepresentation of some phyla in Lake Lanier. The analysis is based on OTU counts, rather than relative abundance, and therefore relates to genetic diversity.

We also identified phyla that are overrepresented in each sub-community of Lake Lanier, using Fisher’s exact test on 2 × 2 contingency tables of target phylum *versus* other phyla, and target SC *versus* other SCs (Fig. 6). This analysis is based on OTU counts, not relative abundance. Therefore, it relates to genetic diversity and not numeric dominance. Sub-communities peaking in months with the highest temperatures (and sunlight), between May and November (SCs 5–11), included OTUs composed 5–12% of photoautotrophic members of the phylum *Cyanobacteria*, with significant overrepresentation detected in SC-10 (peaking in October). In contrast, sub-communities peaking in low-temperature months (January to April; SCs 1–4) displayed a significant overrepresentation of heterotrophic bacteria typically associated with soil or freshwater sediments, including members of the phyla *Verrucomicrobia*, *Bacteroidetes*, and *Acidobacteria*, possibly reflecting less structured communities during the winter resulting in blooms of populations adapted to surrounding environments. OTUs from the phylum *Verrucomicrobia* in SCs 1 and 2 (peaking in January and February, respectively), mainly belonged to the class *Spartobacteria*(SC-1) and the genus *Opitus* (class *Opitutae*; SC-2) or an unclassified member of *Opitutae* (SC 2). A similar pattern was observed among OTUs of *Bacteroidetes* between contiguous SCs 3–4, where members of the family *Chitinophagaceae* (class *Sphingobacteriia*) in SC-3 were succeeded by *Fluviicola* and *Flavobacterium* (family *Cryomorphaceae*, class *Flavobacteria*) in SC-4. Similar trends were observed at the class level (data not shown).

It might be interesting to note that the number of positive *α*_*ij*_ pairs in Lake Lanier is comparable to those in studies of microbial communities growing in human or mouse gut or on spoiling pork^12,22,23^ where organic carbon is presumably abundant. This similarity might suggest that the concentration of carbon sources in Lake Lanier is not the limiting factor for bacterial growth. Of course, the differences between Lakes Mendota and Lanier are multifold, and a single aspect like the organic carbon is not likely to dominate the dynamics in these lakes.

The *α*_*ii*_ values correspond to ratios of birth rates to carrying capacities^13^. These *α*_*ii*_ values are not significantly different among the SCs in Lake Lanier (Fig. S9). By contrast, SCs of Lake Mendota that are blooming during the summer have substantially smaller ratios than SCs in the winter.

The terms *β*_*ik*_ · *T*_*k*_, which characterize the effects of the environmental factors on SC-*i* in Lake Lanier, are in magnitude less than a third of the corresponding *α*_*ij*_ ·*X*_*j*_ terms in Lake Mendota, with median values of 1.59 and 0.47 for |*α*_*ij*_|·*X*_*j*_ and *β*_*ik*_ ·*T*_*k*_ if *m* = 1, and 1.61 and 0.44 if *m* = 3. The interpretation is that other SCs affect a particular SC much more strongly than do the environmental factors per unit of abundance. This finding is qualitatively different from the results regarding Lake Mendota, where the environmental influence was determined to be roughly three times stronger than average SC-SC interactions^13^.

## Discussion

Recent metagenomic sequencing technologies have advanced our understanding of the multiplicity and complexity of OTUs in shared environments. Yet, while we can measure abundances from month to month, interpreting the overall abundance dynamics and its consequences within a complex microbial population is very difficult and suggests the use of computational analysis. We performed such an analysis here with Lotka-Volterra models that are fully dynamic and automatically account for the fact that the relationships between pairs of OTUs are often asymmetric. The models were shown to capture the abundance dynamics of sub-communities within the population of Lake Lanier well. Moreover, the models generate novel hypotheses regarding the effects of OTUs on the growth of other OTUs and regarding the influence of specific environmental factors that follow annual cycles and collectively reflect seasonal changes in environmental conditions^24–27^ and the OTU-OTU interactions.

Judging collectively by parameter values, the SC-SC interactions are stronger in Lake Lanier than in Lake Mendota, whereas the SC-ENV interactions are weaker. This outcome is presumably due to geography, with Lake Lanier experiencing much milder environmental fluctuations than Lake Mendota. In particular, the water temperatures in Lake Lanier are on average 5 °C higher and fluctuations of other resources such as nitrogen and phosphorus are much smaller (Fig. S4 and Table S1).

The models can be used for predictions of consequences of moderate changes in the OTU abundance distribution or in environmental factors. As one will expect, such predictions have to be made with caution. Clearly, black-swan events or drastic changes are not likely to be captured accurately, and changes in factors not explicitly modeled will not be represented reliably, if at all. These limitations are true for any models (see, *e*.*g*.^9^). Nonetheless, the model facilitates thought experiments. For instance, if one of the included environmental factors is moderately perturbed, the model permits predictions that at suggest the direction and magnitude of responses by the lake system. It is at present impossible to validate these predictions experimentally, since the two lake systems are natural. However, among the three environmental factors involved in the model, we were able to validate our predictions of abundance changes *indirectly* when the water temperature in the model was altered for a period of 30 days (see *Supplements*).

Predictions regarding OTUs are especially difficult to validate. Only very few of the OTUs represent organisms that can be cultivated. In fact, over 99% of the OTUs analyzed here represent species that have not been described previously. Even if some OTUs could be cultivated, the results would be questionable, because growing by themselves or with only a few other species would be quite different from growing against a background of thousands of other OTUs. As a consequence, the functional and metabolic properties of most OTUs cannot be reliably inferred from the available 16S rRNA data, which otherwise might support specific OTU-OTU interactions identified by our models. Genome sequences for these taxa are currently being obtained by genome binning techniques. They might provide more appropriate data for interpreting the computationally inferred interactions in the near future. The characteristics of our models with respect to interactions and the effects of environmental factors on the interactions, as well as the population abundance dynamics, are available at http://www.bst.bme.gatech.edu/research12.php.

The dynamic nature of changes in abundance patterns is closely related to important questions regarding the health of the ecosystem. Although a myriad of factors could affect the lake microbiota, our analysis here suggests that incorporating water temperature is necessary, and that the inclusion of pH and sulfate improves the overall quality of the model further, but that other measured factors have minor effects on the SC levels, at least under normal seasonal fluctuations. Of course, this interpretation needs to be considered with caution, as any environmental factors that are closely correlated throughout the year with temperature, pH or sulfate, could be the real drivers of the population dynamics. It is also important to point out that other factors than the three mentioned above might affect some of the OTUs, because our results apply to the majority of the OTUs but not necessarily to all individual OTUs.

One aspect of our analysis addressed the question whether results from one lake system can be translated to another lake system. It turns out that the generic model structure is applicable, but that, unsurprisingly, caution is necessary with numerical extrapolations. An analysis of lakes downstream of Lake Lanier and connected through the Chattahoochee River indicated considerable similarity (Dam *et al*., unpublished). By contrast, Lake Lanier and Lake Mendota exhibit numerous differences, which is understandable, as the two lakes are located in rather different geographical zones and climates. In particular, six chemical and physical characteristics that were measured in both lakes exhibited substantially different levels. While the differences in nitrogen concentrations were reflected in different OTUs capable of using nitrogen sources, we did not observe a similar effect in the case of carbon sources. All things considered, our results suggest that a common model structure can be used, but that the particularities and parameter values must be recalibrated for analyses of different environments.

On the methodological side, we demonstrated here and elsewhere^13^ that Lotka-Volterra models, extended to accommodate environmental factors and combined with appropriate time series data, permit novel insights into complex microbial population systems that at first might appear to be impossible to obtain. Two alternatives to our LV approach could have been a typical graph-based network analysis and a multivariate autoregressive modeling (MAR) approach. We decided against these alternatives for the following reasons. Network models (*e*.*g*.^28–32^) are constructed from correlations that are based on the presence, absence, or abundance of the species across multiple locations or time points. The vertices represent species, while the edges represent either pairwise or complex relationships. Pairwise interactions are typically characterized with a similarity index or a modified Pearson Correlation Coefficient, while complex relationships are derived from regression or rule-based networks^33,34^. While static correlation networks have the capability of addressing potentially very large and complex communities of thousands of species across multiple environments^28,33^, they are limited in that they do not capture potentially important dynamic trends, such as seasonality, and also ignore the “asymmetry” of relationships between species in a sense that the effect of sub-community A on sub-community B is different from the effect of B on A. Because these aspects are crucially important here, we did not pursue this typical network approach.

MAR models^7–9^ provide great flexibility with respect to the inclusion of environmental conditions as well as noise. They also have the advantage over generic nonlinear dynamic models of permitting parameter estimation with methods of linear regression. However, as discussed comprehensively in^9^, the MAR approach constitutes a linear approximation of a system operating close to a stable steady state, which is quite different from the situation we are facing here; other limitations of MAR models are presented in the *Supplements*. At the same time, the parameter estimation strategy proposed for our LV models minimizes the advantage of linear parameter optimization in MAR, because it is linear as well^13,35^. We therefore did not use this approach, but it could be interesting nevertheless to reanalyze our data within this framework, quasi as an independent validation (or refutation) of our results.

The comparison of the two lake populations sheds light on the roles of biological and physico-geochemical factors affecting the dynamics of bacterial populations. Further studies of this nature, with lakes in similar and in different environments, will permit additional comparisons of the dynamic interaction networks that drive the health of lakes. These types of comparisons will eventually guide us toward hypotheses regarding general principles governing the microbial dynamics in lakes which, if validated, may become the foundation for targeted, rational interventions and rescue measures.

## Materials and Methods

### Data

The dataset for this study consists of two subsets. One contains abundance measurements of OTUs, covering 29 time points, collected between July 2010 and October 2015 from Lake Lanier at a depth of 5 m, which corresponds to the well-oxygenated layer of the water-column. These samples were sequenced with a shotgun metagenomic approach (Illumina short paired-end reads; SRA Project PRJNA214105). The description of the complete metagenomes will be reported elsewhere; here we focus on OTUs that were constructed based on 16S rRNA and 18S rRNA gene fragments encoded by individual metagenomic reads. 16S/18S fragments were recovered using Metaxa2 v2.1^36^ after quality trimming using SolexaQA++ v3.1.3^37^ and adaptor-clipping using Scythe v0.991 (https://github.com/vsbuffalo/scythe). Using the Silva database^38^, about 11,000 OTUs were identified with the closed-reference OTU-picking strategy implemented in Qiime^39^ (see *Supplements* and Fig. S1 for details). The Lanier metagenomic data can be found in the NCBI SRA database as part of BioProject PRJNA497294.

Complementing these data are measurements of 29 physical and chemical factors in Lake Lanier, collected by the Georgia EPA^40^ and our team during the same time frame; see *Supplements* for details.

### Model

We use the Lotka-Volterra modeling format, where *X*_*i*_ represents the abundance of one of *n* species, OTUs, or sub-communities (SCs). The interactions between a given *X*_*i*_ and *X*_*j*_, or between *X*_*i*_ and one of *m* environmental conditions *T*_*k*_, are mathematically represented through two-factor terms. The model thus takes the form

$${\dot{X}}_{i}=\mathop{\sum }\limits_{j=1}^{n}{\alpha }_{ij}{X}_{i}{X}_{j}+\mathop{\sum }\limits_{k=1}^{m}{\beta }_{ik}{X}_{i}{T}_{k},\,\,i=1,\ldots ,n.$$

${\dot{X}}_{i}$ is the rate of change in variable *i*, and the parameters *α*_*ij*_ and *β*_*ik*_ indicate the type (sign) and strength (value) of each interaction between pairs of variables. Specifically, the *n* × *n* matrix of *α*_*ij*_ represents the interactions between any two SCs or OTUs, while the *n* × *m* matrix of *β*_*ik*_ is related to the effects of an environmental factor on a SC. A positive *α* suggests cooperative interactions whereas a negative *α* suggests competition.

Details of the model, the parameter estimation procedure, and various analyses are presented in the *Supplements*.

## Supplementary information

Supplementary information

## Data Availability

The data are available at http://www.bst.bme.gatech.edu/research12.php and at http://enve-omics.ce.gatech.edu/data/. The metagenomic data associated with Lake Lanier can be found in the NCBI SRA database as part of BioProject PRJNA497294.
