# Supplementary material for: Model-based Comparisons of the Abundance Dynamics of Bacterial Communities in Two Lakes
Source: Sci Rep. 2020 Feb 12;10:2423. doi: 10.1038/s41598-020-58769-y (PMC7016141; doi:10.1038/s41598-020-58769-y)
Supplement: Supplementary file 1 — Supplementary information [file 41598_2020_58769_MOESM1_ESM.docx]

**Supplements:**

**Model-based Comparisons of the Abundance Dynamics of**

**Bacterial Communities in Two Lakes**

**Phuongan Dam, Luis M. Rodriguez-R, Chengwei Luo, Janet Hatt , Despina Tsementzi, Konstantinos T. Konstantinidis, and Eberhard O. Voit**

**Data Description**

The sequence data consist of measurements at 30 time points with one duplicated sample from Lake Lanier, collected between 7/1/2010 and 10/15/2015. The specific collection time points are shown in Figure 1 of the *Text*. One data point was removed from consideration due to a relative small number of recovered 16S/18S rRNA sequences. Among the remaining 29 data points, the first 24 (Years 2010-2014) were used to construct the model, and the last 5 (Year 2015) for validation. Approximately 80.1± 6.2% of the 16S/18S rRNA sequences were mapped to available databases at 97% nucleotide identity level. In particular, the metagenomes at each time point contain between 6,231 and 28,757 16S/18S rRNA sequences, which can be mapped onto ~11,000 OTUs of the Silva database^1,2^. The 1,400 highest-abundant OTUs account for about 87.2% ± 2.5% of the mapped population; these percentages change throughout the year. The remaining OTUs are low in abundance; the maximum abundance of these is less than 0.1% throughout the year. The identification of OTUs was achieved for Lake Lanier with 16S and 18S sequences retrieved from metagenomic shotgun sequencing, while the 16S data for Lake Mendota were created via 16S amplicon sequencing. As a consequence, there could be slight differences in phylogenetic resolution and taxa coverage, *e.g.*, primer-bias in Lake Mendota samples, and the inclusion of a small percentage of eukaryotes and archaea in the Lake Lanier data, which could affect the comparisons between the two lakes, although not the model results within Lake Lanier.

In order to identify potential taxonomic biases in the composition of the sub-communities of Lake Lanier, we evaluated uneven OTU counts for different taxa between sub-communities using a $\chi^{2}$-test with simulated *p*-value (1e4 replicates) on the contingency table of *SC* x *Taxon*, with Taxon defined at three different levels: domain, phylum, and class. We identified global taxonomic biases in the SCs at all three levels: domain (*p*-value: 0.015), phylum (*p*-value: 2e-4), and class (*p*-value < 1e-4). Similar results were obtained using Fisher's exact test with simulated *p*-value (1e4 replicates): domain (*p*-value: 0.0046), phylum (*p*-value < 1e-4), and class (*p*-value < 1e-4). Lower taxonomic levels were not tested due to the large number of OTUs without defined classification at order level and below.

In addition to these primary data, we used sequence data collected from other lakes that are connected to Lake Lanier by the Chattahoochee River, as a qualitative validation for model predictions regarding pairwise co-abundances between OTUs. These data consisted of 34 time points that were mostly collected during summer and fall of the same time period (results not shown).

Measurements of 29 physical and chemical conditions of Lake Lanier were collected in the same time frame^3^ as our data and analyzed together with corresponding data from the Georgia EPA^4^. Among twelve features retained for our modeling effort, six are shown in Figure S1 in a superposition of measurements between 2010 and 2015. Other conditions were found to be highly correlated to these features or not relevant for modeling (data not shown).


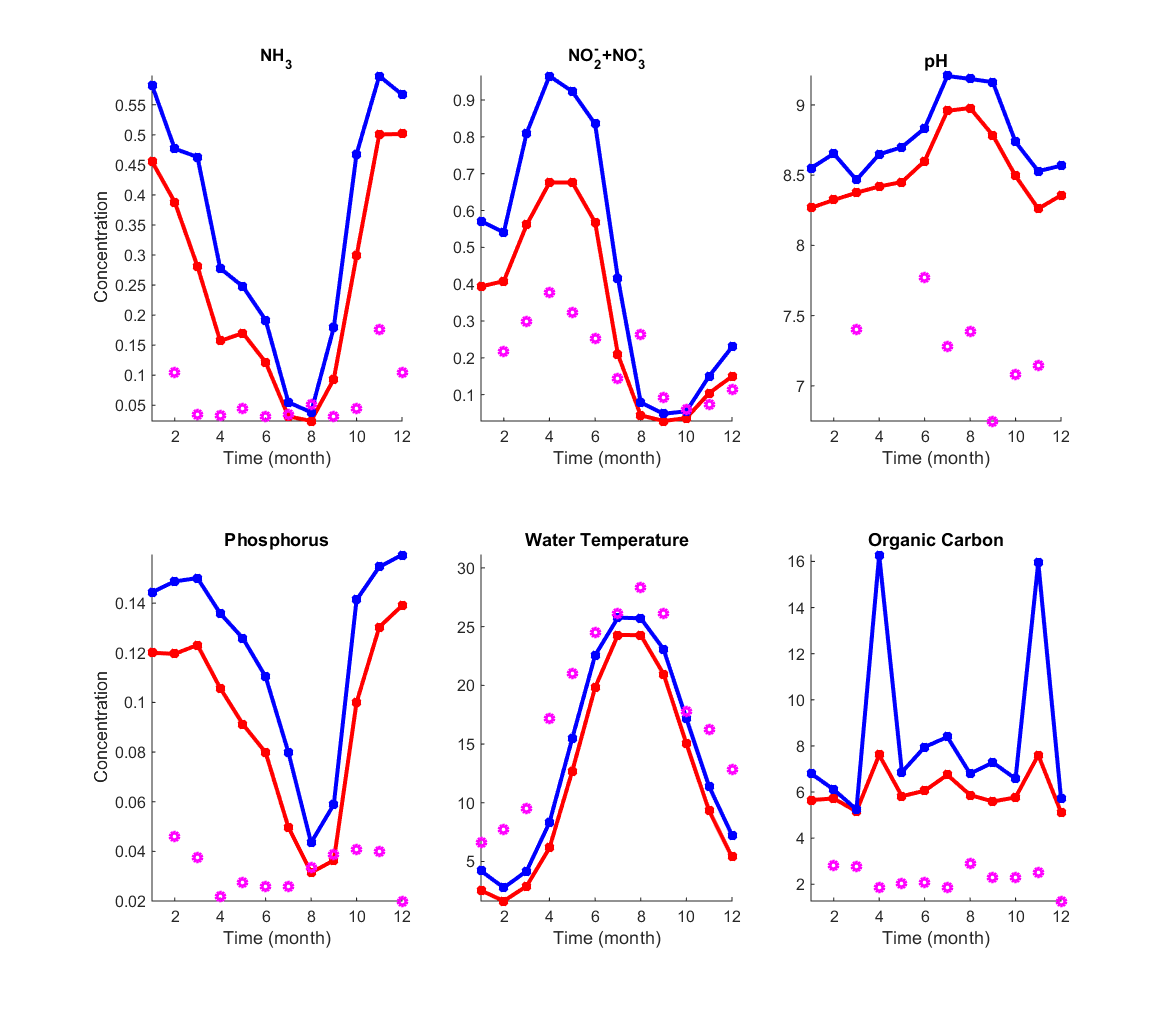


**Figure S1.** **Comparison of chemical and physical characteristics of Lakes Lanier and Mendota.** For each lake, six common chemical and physical characteristics are superimposed into one year. The monthly mean (red) and standard deviation (blue) values computed for Lake Mendota are compared with the means computed for Lake Lanier (magenta dots). The x-axis displays the months of the collective year. Top row from left to right: ammonia (mg/L), nitrite and nitrate (mg/L) and pH. Bottom row from left to right: total phosphorus (mg/L), water temperature (^o^C), and total organic carbon (mg/L).

Lake Lanier has average temperatures between 5.5°C (January) and 26°C (July) with a yearly mean of 16°C. The annual precipitation is 107 cm. Data associated with Lake Lanier are available at <http://enve-omics.ce.gatech.edu/data/>. The metagenomic data can be found in the NCBI SRA database as part of BioProject PRJNA497294 (<https://www.ncbi.nlm.nih.gov/bioproject/?term=PRJNA497294>). Details regarding the results of our analyses are deposited at <http://www.bst.bme.gatech.edu/research12.php>.

Lake Mendota is a drainage lake in Wisconsin, USA. According to the National Oceanic and Atmospheric Administration, its average low temperature is -12.2°C in January and 28.3°C in July with a yearly average of 8.1°C. The average yearly rain and snow falls are 87.9cm and 126.2cm, respectively. Other information can be found at ^5-7^.

**Subsampling and Assignment of Peak Times**

The annual patterns of each OTU were generated with the following scheme. For each OTU, we randomly chose 90% of the data points. These datasets were appropriately superimposed to generate a single, collective one-year period used to establish a representative annual profile. The resulting profile was smoothed by computing the mean values of each moving window of 60-120 days. The position of the maximum value of the profile was recorded and considered as the annual peak time of the OTU. This procedure was repeated 100 times, and the distribution of times (in days) with maximum abundance values for each OTU was constructed. The definite annual peak time of an OTU was defined as the peak of the histogram. Three examples of annual profiles of individual OTUs are shown in Figure S2, where smoothed annual profiles (grey) as well as the median (red) and mean ± standard deviation (magenta and blue) of 100 profiles are displayed. The histograms of annual peak times for 100 profiles are shown in black, and the position of the peak of the (black) histogram was defined as the peak time for the OTU. The second peak of the (black) histogram was identified in the same manner after the first peak was removed.


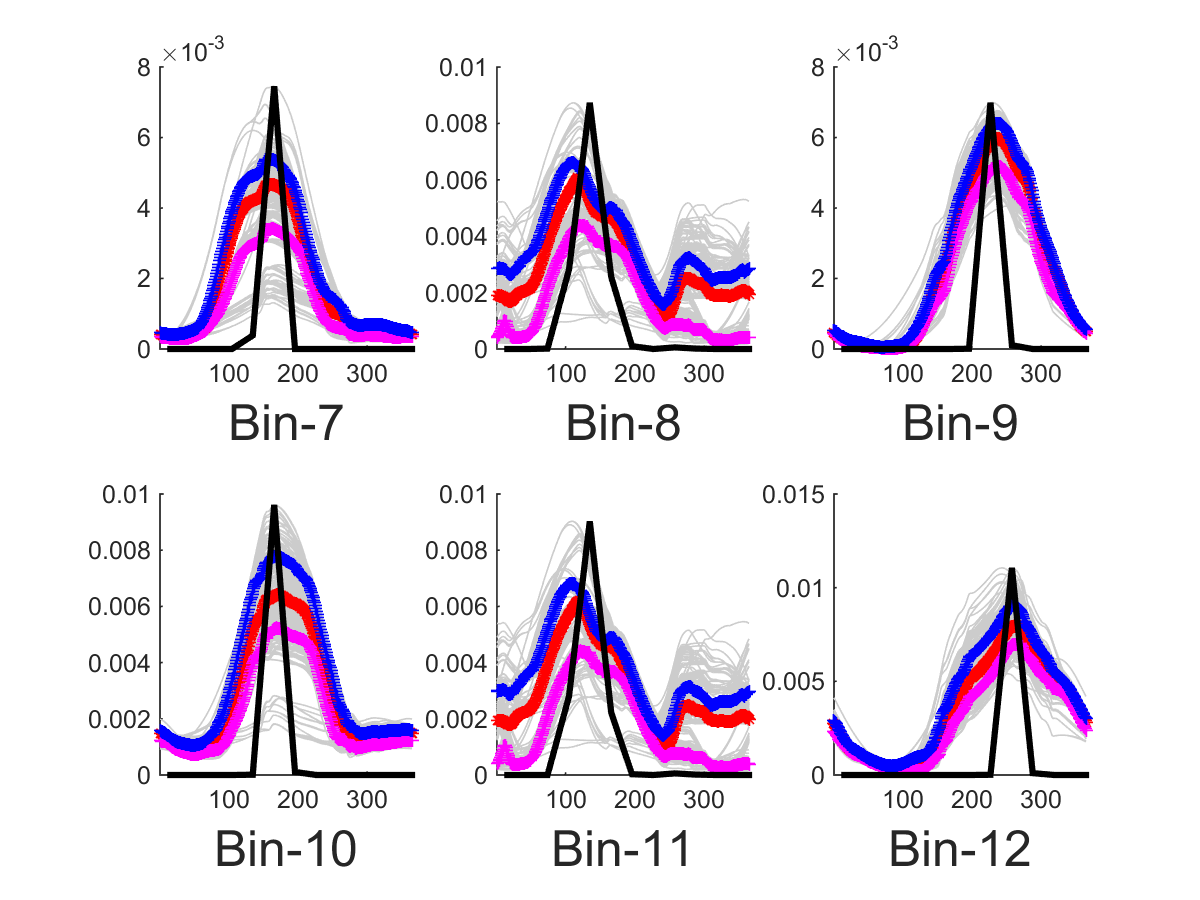


**Figure S2.** **Examples of daily abundances of three individual OTUs in Lake Lanier, superimposed from 2010 to 2015**. Each subplot shows the smoothed profile (grey) of 90% of the abundance data that were randomly sampled, as well as the median (red) and the standard deviation (blue and magenta) of the profiles. The inferred peak time histogram is shown in black. The x-axis represents the day of the year, while the y-axis shows the relative abundance (percentage).

**Definition of Sub-communities**

For each OTU profile, we identified the positions of its top one or two abundance peaks within the year (Figure S2) and then clustered all OTUs based on these peak profiles. This analysis resulted in 13 sub-communities (SCs) with clear peaks plus one additional SC for all remaining OTUs. These 14 bacterial SCs, along with some of the environmental conditions (Figure S3), became the variables in the SC interaction model. As one might expect, the abundances of SCs are quite different in Lakes Lanier and Mendota (Figure S4).


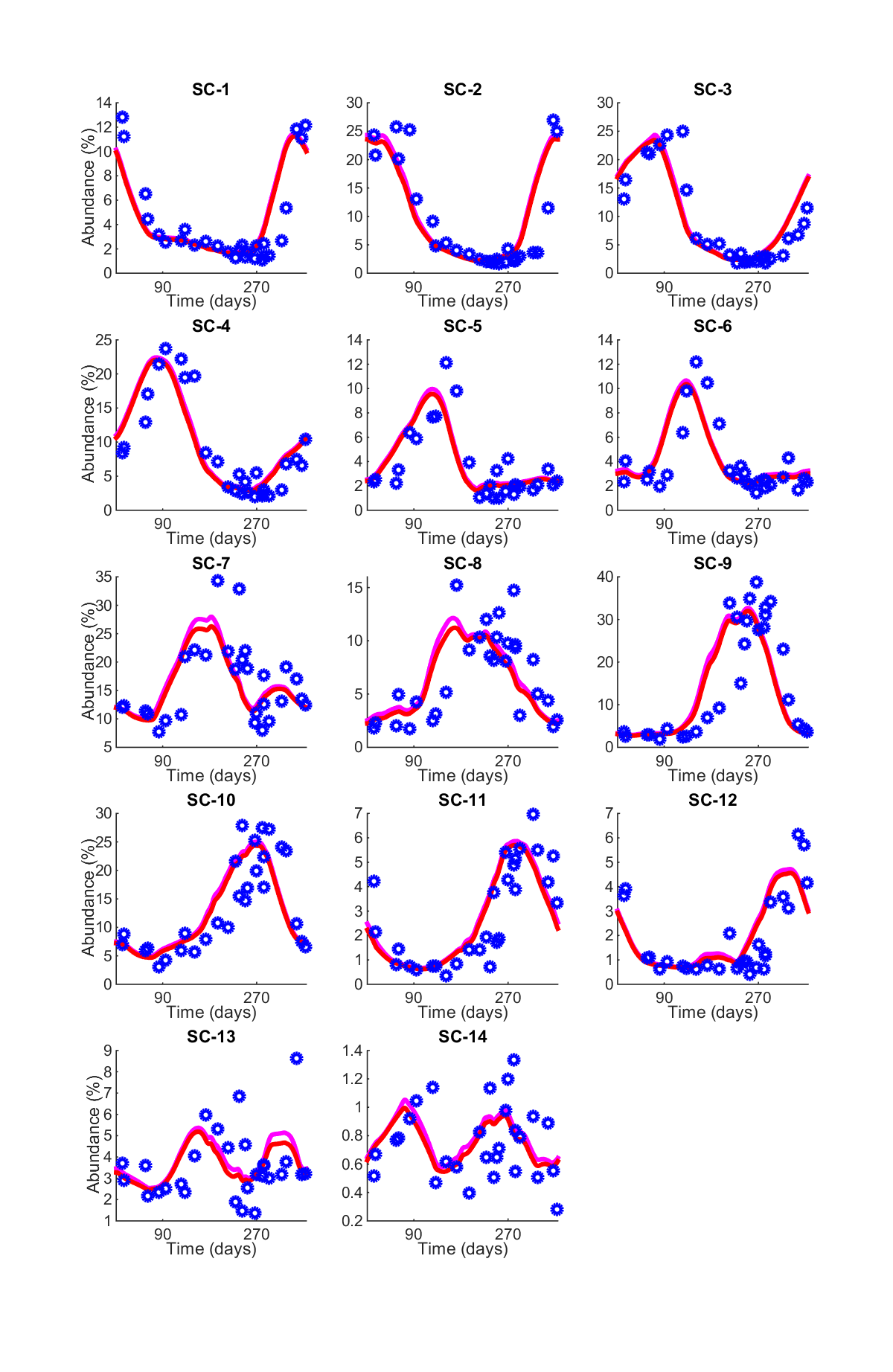


**Figure S3.** **Daily changes in the abundance levels of SCs in Lake Lanier.** The data from 2010-2015 were superimposed into one collective year. The x-axis represents the days in this collective year, while the y-axis shows the total relative abundances (percentage); note differences in scale. The observed data (blue symbols) are shown, along with the average smoothed (red) profile and its standard deviation (magenta), subsampled from original data.


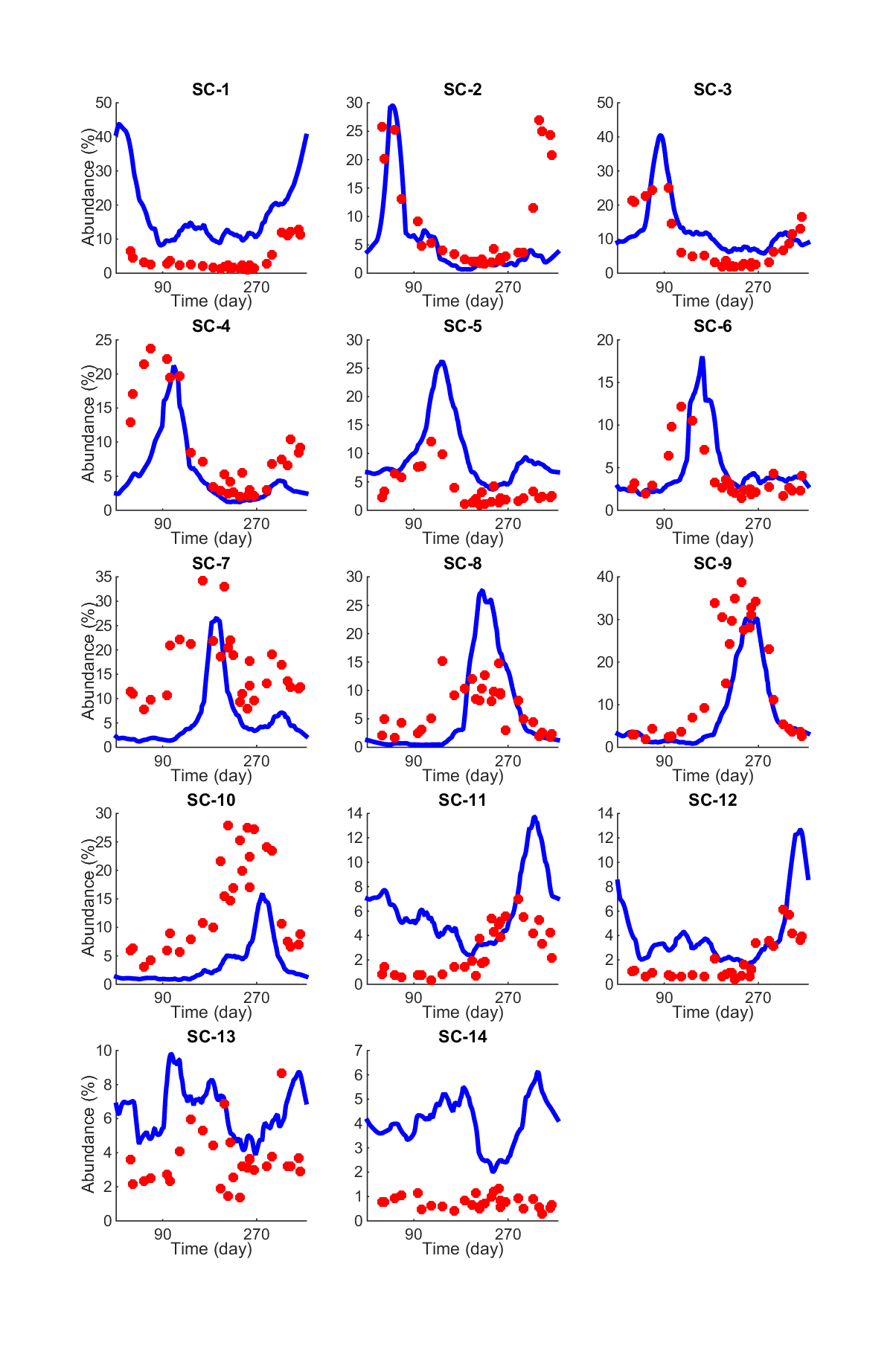


**Figure S4.** **Abundances of SCs in models for Lakes Lanier and Mendota**. The data were superimposed into one collective year and are shown for Lake Mendota as smoothed (blue) lines and for Lake Lanier as red dots. The x-axis displays days of the collective year. The y-axis shows the relative abundances in percent.

**Selection of Environmental Conditions for Modeling**

To test the combination of chemical and physical conditions that most improved the accuracy of predicting the abundance of 14 SCs over time, without unduly increasing the number of parameters, we performed parameter estimations with our Lotka-Volterra model and studied future abundance predictions with all possible combinations (*m*) of up to 12 physical and chemical conditions. We found that accounting just for water temperature in the model (*m* = 1) significantly improved the abundance predictions. Furthermore, adding two chemical and physical conditions, namely pH and sulfate concentration (*m* = 3), significantly improved the prediction. The addition of further conditions did not improve the accuracy of prediction, when the number of parameters was taken into account, suggesting that a good predictive model was achieved with a relatively small number of environmental conditions. To test the significance of adding further conditions, we followed a method for variable reduction by assessing all possible regressions^8^. It should be mentioned that other environmental conditions might be very important, but that they were excluded due to their correlation with one of the three conditions taken into account.

**Model**

We use the Lotka-Volterra (LV) modeling format, where *X_i_* represents the abundance of a species, OTU, or sub-community *i*. The interactions between a given *X_i_* and *X_j_* or between *X_i_* and one of *m* environmental conditions *T_k_* are mathematically formulated through two-factor terms that have their origin in mass action kinetics^9^. The LV model we use thus takes the form

*Ẋ_i_* =$\sum_{j=1}^{n} \alpha_{ij}X_{i}X_{j}$ + $\sum_{k=1}^{m} \beta_{ik}$ $X_{i}T_{k}, i=1, \ldots, n$. (S1)

*Ẋ_i_* is the rate of change in variable *i*, and the indexed parameters *α* and *β* indicate the type (sign) and strength (value) of each interaction between pairs of variables, respectively. A positive *α* suggests a cooperative interaction whereas a negative *α* suggests a (directed) inhibitory or competitive interaction*.*

One should note that the fundamental network structure of typical static models representing microbial communities is retained in the dynamic LV model. However, the dynamic model adds genuine value in several aspects. First, the interactions in the dynamic model are not symmetric, as described in the *Text*. Second, the network structure (*i.e.*, the presence or absence of significant edges) changes dynamically with the seasons, which is captured by dynamic, but not static models. Third, although the dynamic model does not distinguish between direct and indirect interactions, it captures the overall net effects for each pair of OTUs or SCs (in both directions). Specifically, the interaction parameters $\alpha_{ij}$ reflect the totality of effects of one particular OTU (or SC) A on another OTU (or SC) B. Similarly, the corresponding $\alpha_{ji}$reflect the totality of effects of B on A. It is quite possible that these interactions are mediated by, for instance, molecules excreted by some third OTU or by a higher-order consumer.

An alternative to our LV approach could have been the use of multivariate autoregressive (MAR) models (*e.g.*, ^10,11^), which have been shown to capture certain features of ecological communities well. However, we did not pursue these models for several reasons, many of which can be found in^12^.

1. MAR is a local, linear approximation of a typically nonlinear population interaction system. Because our LV approach addresses the nonlinear system directly, even if in a simplified form, it is hard to see how the MAR approximation could improve our strategy except, importantly, in a methodological manner and, in particular, the fact that the linearity of MAR is a clear advantage over nonlinear parameter estimation approaches. However, by using the parameter inference technique we propose (see below), we are able to convert the originally nonlinear optimization task into a linear regression problem, so that the advantage of linearity on MAR is moot in our case.

2. Several groups (see ^12^) have warned that linear approaches such as MAR may simply be inappropriate to model nonlinear ecological dynamics. We are able to exploit the full dynamics, as long as it is more or less appropriately captured by the Lotka-Volterra representation.

3. MAR was designed as an approximation close to a stable steady state and runs into problems even for two-variable LV systems that oscillate ^12^. A quick look at our data makes it clear that the bacterial communities in the lakes we study are far from operating close to a steady state and oscillate with wide amplitudes..

4. As a recursive approach, MAR explicitly accounts for noise, which is problematic for ODE models, lest the complexity skyrockets. However, the noise in MAR is explicitly taken as normally distributed, with zero mean and moderate variance, which may or may not be true in systems acting more on a logarithmic than a linear scale. Furthermore, the noise in MAR models is typically subsumed into the environmental effects matrix and therefore explicitly dropped. The result is therefore quite similar in concept, although not mathematics, to our approach

5. Inferences from MAR are relatively robust with respect to the signs, but not magnitudes, of the interaction parameters. Interestingly, we have come to the same conclusion (see *Text*) and taken great care not to interpret anything into the magnitudes. Instead, our comparison of lakes reveals different profiles of signs, that is, of competitive and collaborative interactions.

6. Due to the approximate nature of MAR, parameters are conglomerates of different features and their values should not be interpreted mechanistically. This feature is similar for our LV models, in which the “interaction parameters” collectively represent direct as well as indirect interactions. We do not believe that anything else can be done with the available data to tease these effects apart.

7. Certain *et al.* ^12^ warn that “MAR(1) models are better at making short-term qualitative forecasts (next point going up or down) than at predicting long-term responses to environmental perturbations, which can be severely over- as well as underestimated.” This prediction risk is no surprise and similarly applies to our models. In his seminal paper on MAR modeling, Ives^10^ warned that even modestly complex systems present “a considerable challenge to the [MAR] prediction techniques.” The future is simply impossible to predict if black swan events occur; specifically, MAR predictions only seem reasonable if the means of the stationary distributions of species abundances and environmental covariates remain constant throughout time. We are in a similar situation with our LV approach where predictions should be limited to moderate perturbations in conditions or other features that are explicitly represented in the model.

Finally, MAR model require long time series (see following section), which do not currently exist, at least not for our lake systems. In spite of these issues, it could be interesting to reanalyze our data with MAR methods and to compare results with ours.

**Parameter Estimation**

One of the significant advantages of MAR models over generic nonlinear dynamic models is the fact that parameter values can be estimated with methods of linear regression. Two issues reduce this advantage in our case. First, upon an equivalence transformation, the LV model permits parameter estimation with linear regression methods (see below and ^5,13^). Second, Certain and colleagues^12^ recommended that the inference of interaction parameters in MAR models should be based on time series that are at least five times as long as the number of parameters to be estimated, just to infer the signs (not even the magnitudes) of interactions. Our system has roughly 250 parameters (including the effects of the three representative environmental conditions; 14 × 18), which would require 1250 monthly time points; in other words, 100 years.

Of course, it would be desirable to base the modeling on much longer and denser time series than we have available, especially for the estimation of parameter values. However, these types of data are expensive and therefore scarce. Our previous study on Lake Mendota, which was based on more comprehensive data, allowed us to test and calibrate the various methodologies we are using here, and to compare and contrast them to alternative approaches. This earlier analysis and the analysis here indicate that the results are quite robust, even for the sparser data from Lake Lanier. Importantly, we are not relying on singular data fits or parameter values, but on *ensembles* of model parameterizations, which we then interpret collectively, for instance, with respect to patterns in positive and negative parameter values (see below). As a consequence, it appears that our results are solid and that they yield comparative insights into two quite different lake environments that had not been gained by other methods. Specifically, our estimation effort followed the strategies outlined below.

For each regression task, we ignored the situation *X_i_* = 0, which here is irrelevant. Consequently, Eq. (S1) can be rewritten as

$\frac{⨰_{i}}{X_{i}}$ =$\sum_{j=1}^{n} \alpha_{ij}X_{j}$ + $\sum_{k=1}^{m} \beta_{ik}$ $T_{k}, i=1, \ldots, n.$ (S2)

The strategy for analyzing this equation is to estimate both abundances and slopes, directly from the time courses of all SCs and ENVs. Substituting these quantities into Eq. (S2), the model becomes a system of linear algebraic equations, and constrained linear regression with a function like *lsqlin* in Matlab can be used to solve for all parameter values ^14-18^. As objective function we used the traditional sum of squared residual errors (SSE). This SSE covers all data points used in each fitting; the quantities to be altered toward minimization of SSE are the parameter values.

A well-recognized issue with parameter estimation based on slopes is that the determination of slopes from noisy data is challenging. However, this strategy enormously simplifies the task at hand and makes it much more robust. As a demonstration, we previously clocked the time for the slope-based strategy and compared it with traditional estimation techniques directly based on nonlinear differential equations^15^. The slope-based strategy used on average only between 1% and 5% of the total time the nonlinear methods used. In other words, the time saving amounted to over 95%. In addition, the method used here does not become stuck in local minima and does not run into issues with stiffness, which some parameter combinations can create. In cases of stiffness, the savings in time approach 100% (see^15^).

While the advantages of the slope-based method are appealing, parameter estimation remains to be challenging. We deal with the situation in two ways: First, the LV estimation problem uses regular linear regression^14^. This fact is important, as linear regression tasks have explicit solutions and do not become trapped in local minima, as nonlinear tasks tend to do. Notwithstanding, the unique solution of a linear regression task is quite sensitive to noise in the data. We therefore constrain the parameters $\alpha_{ii}$ to different values (see below) and, in doing so, create thousands of vectors of parameter values, of which we retain an *ensemble* of up to dozens or a few hundred solutions that fit the data with similar, particularly low residual errors. All our subsequent interpretations are based on patterns within these ensembles, rather than singular optimized values. This strategy makes our results much more robust than singular optimal set of parameter values.

Specifically, we estimated the abundances and slopes of each SC (in the 14-SC model) or individual OTU (in the OTU model), we used the midpoint for each pair of consecutive data points. For the estimation itself, we constrained the upper bound of the $\alpha_{ii}$ values to some value within the range [-1, 0]. The rationale for this choice was that this parameter appears in the standard logistic growth model as the so-called crowding effect. Specifically, –$\alpha_{ii}$ is proportional to the intrinsic birth rate, divided by the carrying capacity, and must therefore be positive. Also, positive values for $\alpha_{ii}$ tend to destabilize the system.

Ensembles of close-to-optimal parameter values were obtained through very many constrained linear regressions, where the $\alpha_{ii}$ values were differently selected every time. Theoretically, infinitely many combinations of these $\alpha_{ii}$ are possible, and to establish a reasonable ensemble, we iterated each regression task *R* times, where *R* is set as 200 times the number of parameters that were to be estimated in the given task. Thus, *R* values for different tasks ranged in the thousands. From among the results of these thousands of regressions, we selected ensembles of those parameter sets that were very close to optimal. For *m*=1, which means that we only considered temperature as a possible confounder, 193 solutions turned out to be close to optimal, *i.e.*, were very similar in minimized SSEs. For *m*=3, indicating the inclusion of temperature, pH and nitrate concentration, 84 solutions had similar, minimized SSEs. Parameter values estimated for the Lake Lanier model are presented in Tables S3 – S5.

To predict changes in abundances during simulated disturbances of environmental factors, we used a modified least-squares (LSQ) metric. Specifically, we defined LSQ=Σ((*y_d_*-*y_o_*)/mean(*y_o_*))^2^, where *y_d_* is the abundance during the disturbance, as predicted by the model, and *y_o_* is the original abundance in the unperturbed system.

**Similarity Scores**

As in our previous study^5^, the quality of the results from each model was assessed by a combined similarity score, which we defined as

*Score* = 1 - *S_p_* + *S_w_*. (S3)

Here, *S_p_* is the Pearson correlation coefficient (PCC) between the predicted and the observed abundance, and

*S_w_* = min(abs(log_2_($\frac{{\sum{(y}_{pred}{-(min(y}_{obs})))}^{2}}{{\sum{(y}_{obs}{-(min(y}_{obs})))}^{2}}$)), 2) (S4)

is a measure of the difference between the observed and predicted peaks of a sub-community.

The goodness of fit was evaluated with the PCC score (*S_p_*) for the data and the predicted value, as well as the fitness score (S_w_) shown in Eq. (S4). A model parameterization was judged to be of “high quality” if *S_w_* <= 1 and *S_p_* >= 0.5, and of “good quality” if *S_w_* < 2 and S_p_ > 0.

**Dynamic Model Predictions of SC Abundances**

It is of interest to assess the importance of accounting for environmental conditions in the model further. As an illustration, consider the best models within two categories, A and B, where model A contains 14 SCs and just water temperature, whereas model B contains 14 SCs as well as water temperature, pH and sulfate concentration. While the two models obviously have different parameterizations, it is intriguing that the positive or negative trends in the *α_ij_* values are quite consistent throughout the two models (Figures S5-S6). It is interesting to note that the signs, but not necessarily the magnitudes, of interaction parameters are also quite robustly estimated with other modeling approaches, such as MAR^12^. Moreover, the means and standard deviations of all *α_ij_* values in our analysis do not exhibit significant differences between the two models in 85.7% of all *α_ij_* pairs (including *α_ii_*). These results suggest that the estimated *α_ij_* values, which indicate competition or cooperation, are relatively stable even with the addition of new environmental factors, and that the two chemical features included in model B directly or indirectly improve the overall fit. Interestingly, SC-1 and SC-13, which peak in the winter, have the highest number of *α_ij_* values (35.7%) that are significantly different between the two models, while SC-5, SC-6 and SC-9, which peak in spring and fall, show no significant differences in *α_ij_* values between two models. Our observation suggests that the addition of pH and sulfate improved the abundance prediction for SC-1 and SC-13. Neither the pH level nor the sulfate concentration shows a consistent annual pattern. These observations suggest that the effects of environmental conditions could be overshadowed in the model by the effects of the 14 SCs, although such effects could still be present in the lake.


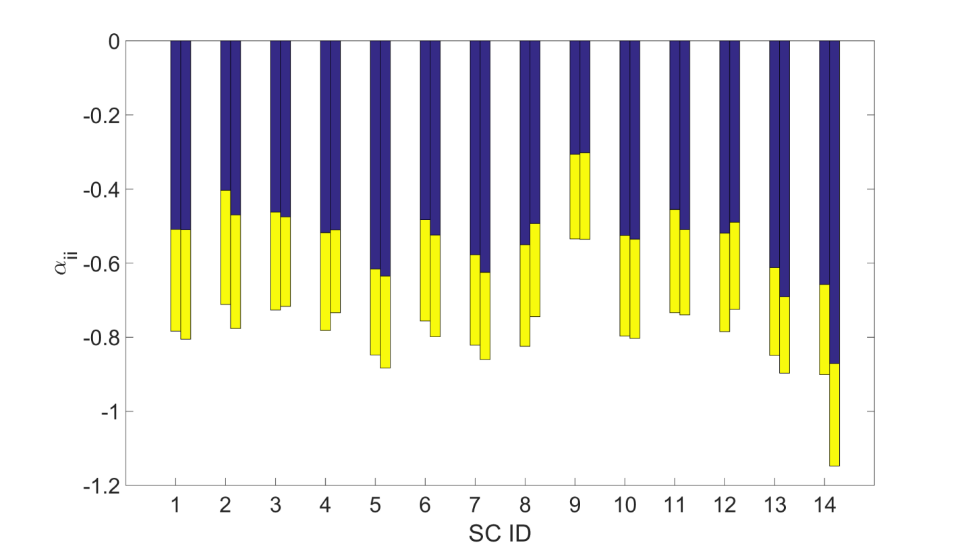


**Figure S5.** **Estimated *α_ii_* values in ensembles of differently parameterized models of the 14 sub-communities in Lake Lanier**. The bar plot shows *α_ii_* values of SC1 to SC14 for two models with *m*=1 (left) and *m*=3 (right) environmental conditions included (see Text). For each model, 193 (*m*=1) or 84 (*m*=3) parameterizations were used for the analysis. The means (blue) and the standard deviations (yellow) of the *α_ii_* values are not significantly different among SCs.

**Effects of Environmental Disturbances**

Our analysis demonstrated that a change in water temperature causes a larger change in the predicted abundances of SCs if the change happens at some time between January and May rather than between June and December (Figure 5B). While it is impossible to validate this prediction directly, an indirect validation is possible with the following approach: We divided the abundance data into two groups based on blooming time. One group included all data collected from January to May, while the other included the remaining data. For each monthly abundance measurement in each of the 14 SCs, we computed the difference between the actually observed abundance value and the corresponding average value derived from the period 2010-2015. Similarly, we computed for each month the difference between the observed temperature and the averaged water temperature for this month. From these quantities, we computed the ratio of the difference in abundance over the difference in water temperature. ANOVA for this comparison revealed that the average change in observed abundances of the 14 SCs per unit of change in water temperature is significantly different between the two groups of months (*p* = 0.03), and that the effect is significantly stronger in the spring compared to the rest of the year.

**Comparison of Interaction Terms (*α_ij_*) in the Models of the Two Lakes**

We compared the *α_ij_* values of the top 200 instances of the SC-SC model of Lake Mendota and 84 instances of the SC-SC model of Lake Lanier, as shown in Figures S6. In all cases, the |*α_ij_*| values are larger in the model for Lake Lanier, indicating stronger interactions. In addition, we found that 50% of the median values have the same sign (positive or negative effect).


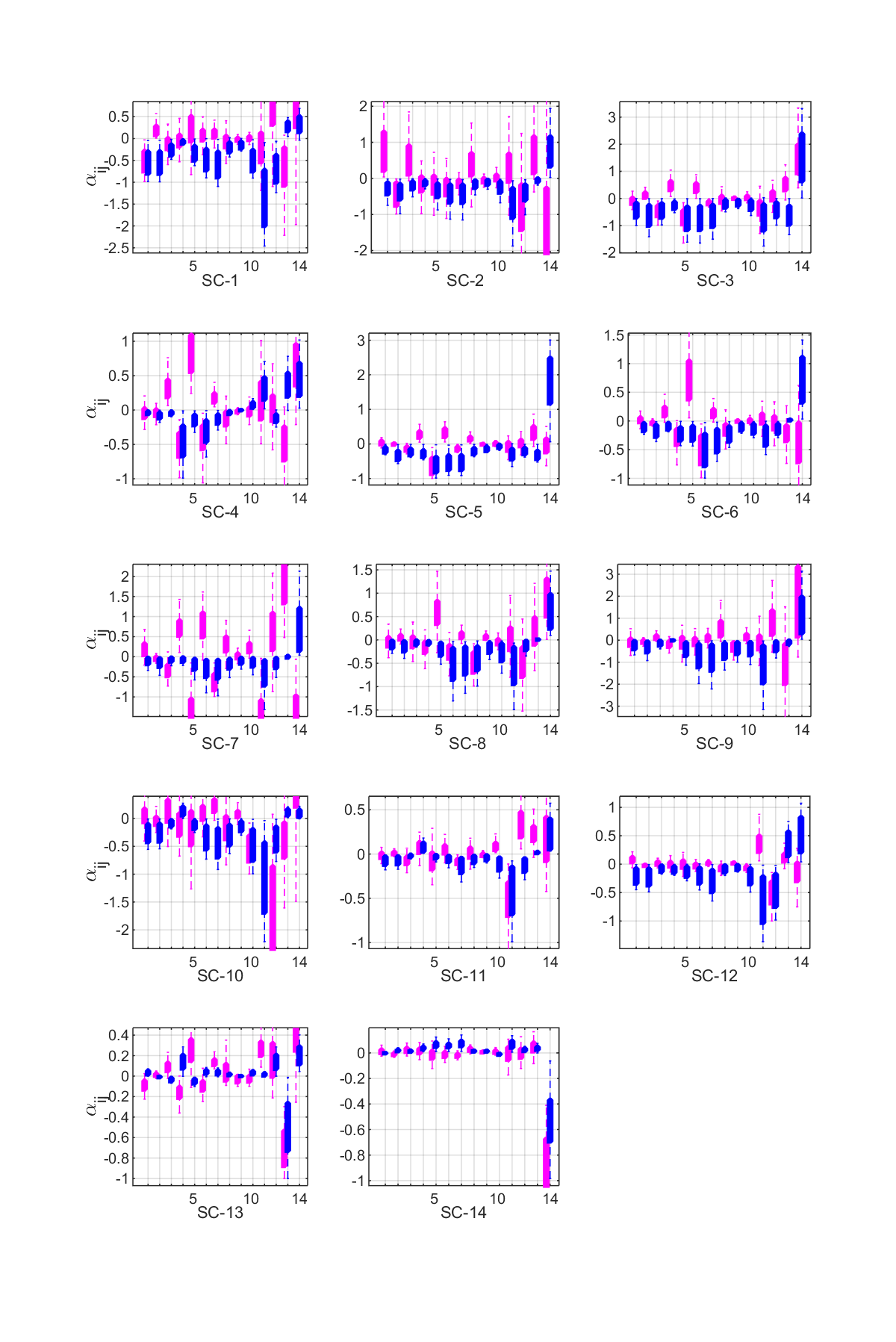


**Figure S6.** ***α_ij_* terms in ensembles of differently parameterized models of Lakes Lanier and Mendota**. The *α_ij_* values for the top 84 and 200 instances of models, respectively for Lake Lanier (magenta) and Lake Mendota (blue), are shown. Each sub-boxplot shows the range of pairwise interaction parameters *α_ij_* of a given SC on the SC displayed along the x-axis. The subplots show that the *α_ij_* values of the two dynamic models are not the same.

**Abundances of Individual OTUs and their Interactions**

After exploring the base LV model for different combinations of environmental conditions, we analyzed models of the same mathematical format, but with variables representing individual OTUs. As described in a previous study^5^, most OTUs within the same SC follow the general trend of the abundance pattern of the SC; however, some OTUs display characteristics that can only be explained through models at the individual OTU level. For example, our previous study suggested that almost 90% of the OTU abundance predictions can be further improved in this step, and some OTUs have smaller or larger estimated $\alpha_{ii}$ or $\beta_{ik}$ values than predicted in the general SC-14 model. In addition, many individual OTUs were predicted by the model to be strongly effected by environmental conditions, which was supported by experimental data^5^.

For example, in the dynamic model of Lake Mendota, OTU#141903, a member of the family *Nitrosomonadaceae*, was predicted to be affected strongly by ammonia. Indeed, previous studies suggested that all cultivated representatives of this group are able to oxidize ammonia^19^, which is consistent with our results. Similarly, OTU#517152, a member of the genus *Roseomonas*, was predicted to be affected positively by water temperature. Various members of this genus have been found in aquatic environments. They were described as growing better at 25-28^o^C^20^ than in colder water and in some cases thrive up to 42^o^C^21^. The same procedure was applied to OTUs found in Lake Lanier. The modeling results of the top 100 most abundance OTUs in Lake Lanier is reported in our websites (<http://www.bst.bme.gatech.edu/research12.php>) and at <http://enve-omics.ce.gatech.edu/data/>).

Using the model for the 14 SCs, we assessed the abundances of individual OTUs in Lake Lanier (Figure S7). Among the top most abundant 1,000 OTUs, the abundance patterns of 687 OTUs were predicted successfully from the general 14-SC model (results are reported at our website). Upon closer examination of the total relative abundance of all OTUs within an SC, which was predicted well among the top 1,000 OTUs, we found that OTUs belonging to SC-14 and SC-9 were not predicted as well as other SCs. In particular, by abundance, only 55.4% and 74.2% of SC-9 and SC-14 have high-quality models according to our criteria. In the case of SC-14, the slightly inferior predictability is understandable because SC-14 contains all those OTUs that do not exhibit well-defined annual peaks. The lower predictability for SC-9 is more difficult to explain. The model does offer good predictions for 55.4±8.9% of the total abundance of SC-9, but it seems that other factors, which are unknown and were not taken into account, are important for the remaining OTUs.

**Co-abundances between OTU Pairs**

To predict the effect of any OTU-A on another OTU-B, we performed linear regression either between *X_i_* and *X_j_* or between $\dot{X}_{i}$ and $X_{j}$ for each period where $\dot{X}_{i}$ is increasing or decreasing monotonically. The *p*-value cut-off for predicting an interaction pair was Bonferroni-corrected. For each pairwise interaction with significant *p*-value, we validated the result using the data collected from other lakes connected to Lake Lanier (data not shown). For the linear regressions between each pair of *X_i_* and *X_j,_* among the ~11,000 OTUs, we found 100 pairs with significant *p*-values in data collected from Lake Lanier. The 100 significant pairwise co-abundances involve 97 OTUs whose abundance ranges from 25.2-46.9% of the mapped OTUs from Lake Lanier. Among these, 49 OTUs belong to seven large clusters of four or more members, and the remaining OTUs belong to 21 small clusters of two or three members (see Tables S4a and S4b for details). Some results were highlighted in the Text. Others follow below.


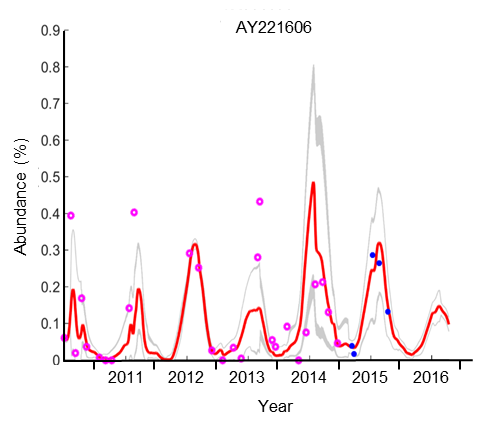


**Figure S7**. **Observed, modeled, and predicted annual abundance of an individual OTU (named according to the Silva database) in Lake Lanier.** The abscissa shows the six-year period starting from 7/1/2010, and the ordinate shows the relative abundance (percentages) of the OTU; tick marks indicate beginning of years. The observed values were collected between July 2010 and December 2014 (magenta). They were used for parameter estimation. We used each observed data point (magenta) to predict the abundance of the OTU for the next 365 days, using 10 randomly chosen instances among 84 instances of the model (*k*=3). The median (red), maximum and minimum (grey) of the predicted values are shown. Data collected from March to October 2015 (blue) were used as validation. The results suggest that future abundances of an OTU can be predicted based on past observations.

The largest cluster (#1) contains 11 OTUs from the family *Planctomycetaceae*. These 11 OTUs account for 58% of the 16S sequences assigned to this family in our data sets. Species within this family have been reported to tolerate extreme environments such as cold or acidity stress^22^. In Lake Lanier, these OTUs peak in late summer, suggesting that they prefer warmer temperatures.

The second largest cluster (#2) contains ten OTUs in three genera, namely *Novosphingobium* (1), *Ralstonia* (2), *Polynucleobacter* (6), and the order *Rhodocyclaceae* (AB255118). Previous reports suggest that members of the genus *Novosphingobium* are aromatic compound degraders using substrates like phenol, aniline, and nitrobenzene^23^. Members of the genus *Ralstonia* are oligotrophic and can survive at very low nutrient concentrations^24^. Members of the genus *Polynucleobacter* are either free living or symbionts in fresh water^25^, and these six OTUs comprised 63.2% of the total *Polynucleobacter* in our datasets. The order *Rhodocyclaceae* is more diverse, and its members can be anoxygenic photoheterotrophs, aerobes fixing plant-associated nitrogen, or utilizers of aromatic compounds, oxygen, nitrate, chlorate, perchlorate selenate, sulfur and others^26^.

The third largest cluster (#3) contains nine OTUs from three families including *Flavobacteriaceae* (1), *Comamonadaceae* (6) and *Oxalobacteraceae* (2)*.* The literature suggests that four out of nine members of this group can utilize nitrogen compounds^27-30^. Interestingly, the high abundances of these OTUs co-occur in Lake Lanier with low water temperatures and high levels of nitrate. Furthermore, recent literature documents that the remaining members of this group belong to genera whose members have recently been shown either to utilize nitrate or contain genes for this capacity.

Cluster #5 contains five OTUs from two groups including the clades SAR-11 (4) and CL500-29 (1). SAR-11 is highly abundant in oceans, where it utilizes dissolved organic carbon and nitrogen, thereby compensating for its inability to fix carbon or nitrogen^31^. In our data, the four SAR-11 OTUs comprise 76.8% of the total SAR-11 cells and 5.7% of the total abundance of collected DNA sequences. During the last three years of data collection (2013-2015), the peaks of the four SAR-11 OTUs coincided with the peak of SO_4_^-^. A literature search revealed that *Pelagibacter ubique*, a member of this group, has an unusual requirement for reduced sulfur^32^ and cannot assimilate SO_4_^-^. Previous studies suggested that the requirement for exogenous reduced sulfur is common, if not even universal, for bacteria in the SAR-11 group, and implied that the source of the reduced sulfur should come from other microbes. The remaining member of this cluster is OTU AY466491, which is assigned to the family *Acidimicrobiaceae* and the genus *Ilumatobactor* (RDP). Two known members of this genus have been sequenced; their genomes contain genes belonging to the sulfate assimilation pathway, which however seems to be incomplete (BioCyc).

Cluster #6 contains four OTUs in two groups including the order *Burkholderiales* (3) and an uncultured proteobacterium (OTU FJ612419) assigned to the genus *Roseomonas* (http://rdp.cme.msu.edu). These OTUs have diverse phenotypes, but we found that they are all highly correlated to the water temperature. Interestingly, previous study suggested that the *Roseomonas lacus sp. nov.* strain TH-G33 collected from a freshwater lake grows optimally at 30^o^C ^33^, while many other *Roseomonas* species are human pathogens^21^.

Finally, one interaction cluster contains three OTUs in two taxonomical groups from the class *Opitutae* (AY863090 and AY752112) and the genus *Shingomonas* (AJ575707). Some members of the *Opitutae* degrade heteropolysaccharides^34^, while the genus *Shingomonas* produces exopolysaccharides and utilizes diverse carbon sources including polycyclic aromatic compounds^35^. Interestingly, AY863090 and AY752112 are classified in the RDP database as belonging to genus *Cerasicoccus*. Members of this genus utilize arabinose and cellobiose. The abundance of AY752112 can predict the abundance of AY863090 (data not shown), but the abundance of the other two OTUs cannot be predicted by their cluster partners, which suggests that AY752112 is likely to affect the growth of AY863090, possibly through pathways of heteropolysaccharide degradation.

**Seasonality of the Microbial Communities in Lake Lanier and Lake Mendota**

After assigning the peak time to each individual OTU, we tested if its observed abundance during peak time was significantly different from other time points. Specifically, we combined the time series data of all years, determined a blooming period for each OTU, and tested if the abundances of the OTUs during the blooming period were significantly different from the remaining time period. Using blooming periods varying from 30 days to 180 days, and a *p*-value cut-off of 0.05, each OTU was declared to have a significant abundance difference during its peak time if the *p*-value of one of the *t*-tests was significant. We found that 5,620 (51.8%) of the mapped OTUs had abundances around their peak times that were significantly higher than abundances collected at other time points. These OTUs account for 77.6 ±6.8% of the annual abundances of all mapped OTUs.

When the OTU peaks in Lake Lanier were categorized by their season or month, significant differences were found. The average lengths of computed OTU blooming times, grouped by month, are presented in Figure S8. The length of the peak times for OTUs blooming in the summer (June to August) turned out to be shorter than for other seasons (67.8 versus 79.0.0-81.6 days). In particular, the lengths of the peak times for OTUs blooming in June turned out to be the shortest (Figure S8). This difference was significant in comparison with other months (61.2 versus 74.0-86.6 days, *p*-value of 5.12E-10). By contrast, the lengths of the peak times for OTUs blooming in March were significantly longer than for June and December (86.6 versus 61.2 and 74.0 days).


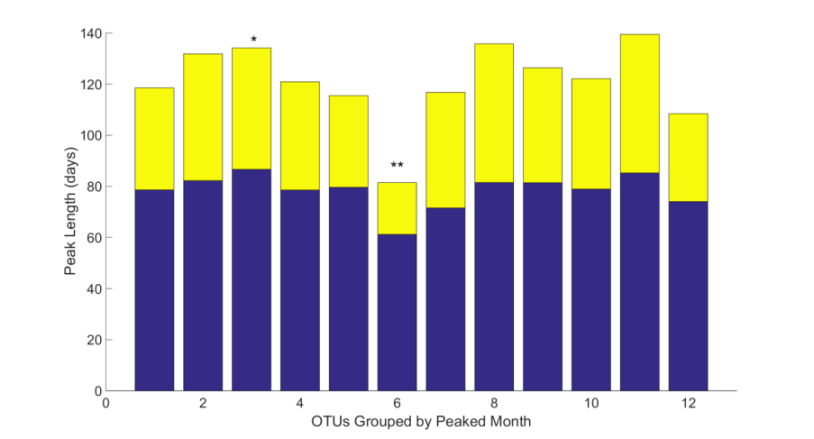


Month

**Figure S8.** **Inferred lengths of blooming periods of OTUs in Lake Lanier, grouped by SC.** The x-axis represents the label of the SC, while the y-axis shows the average length (blue) and the standard deviation (yellow) of significant peaks. (**) OTUs peaking in June (SC-6) have shorter blooming periods compared to other months; the differences are significant except for July. (*) OTUs peaking in March (SC-3) have significantly longer blooming period compared to June (SC-6) and December (SC-12). SC-13 and SC-14 were not included because the peaks in these SCs are not significant.

In addition to exclusively focusing on bacterial data, we found a significant difference (*p* = 5.12E-10) between the lengths of the predicted blooming periods of bacteria and microscopic eukaryotes (based on 18S rRNA gene-encoding reads) in Lake Lanier. Specifically, the average blooming periods of archaea, bacteria and microscopic eukaryotes are 58.1±10.7, 79.6±0.6 and 64.5±2.3 days, respectively (see Table S7 for details). This analysis suggests that the blooming period of bacteria is significantly longer than for microscopic eukaryotes, implying that either the latter organisms are consumed faster or are less resilience while the former are more resilient to seasonal changes in the environment.

In a comparison between lakes, 22.1% and 36.9% of the OTUs in Lake Lanier have blooming periods of 30 or 60 days, respectively (Figure S9). By contrast, 81.2% of the mapped OTUs in Lake Mendota have significant blooming periods, and for 68.2% and 21.1% of these the respective blooming periods last for 30 or 60 days. These results demonstrate that a larger proportion of OTUs in Lake Mendota exhibits more intense annual patterns, and that the high abundance period is shorter than for OTUs in Lake Lanier. The predicted blooming times and lengths of these (~10,000) OTUs can be downloaded from our website (<http://www.bst.bme.gatech.edu/research12.php>).

**
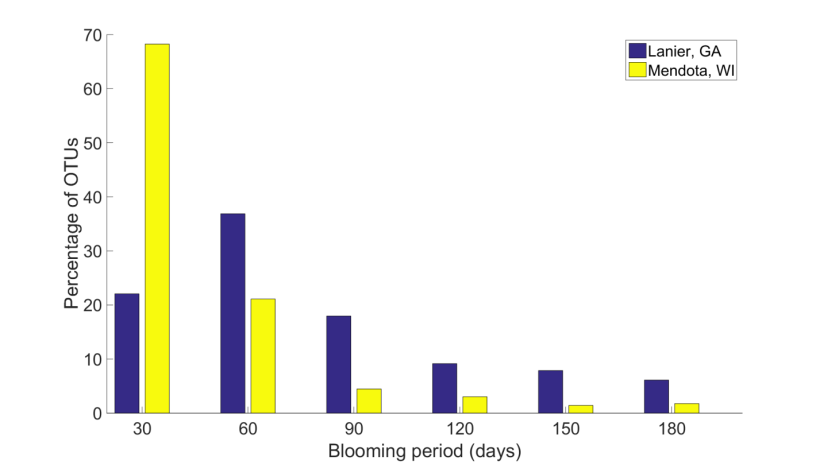
**

**Figure S9.** **Distribution of lengths of blooming periods of OTUs in the two lakes.** The x-axis represents the length of the blooming period in days, while the y-axis shows the percentage of the mapped OTUs with significant peaks. Data were collected from Lake Lanier, GA (blue) and Lake Mendota, WI (yellow). The results demonstrate that the blooming periods of bacterial species in Lake Mendota are on average shorter than in Lake Lanier.

A comparison of the mean blooming periods computed for similar taxonomical families present in Lake Lanier and Lake Mendota reveals that they are correlated (Figure S10). The six bacterial families whose peak lengths are most similar between the two lakes are *Chitinophagaceae*, *Ruminococcaceae*, *Chthoniobacteraceae*, *Lachnospiraceae*, *Rhodospirillaceae*, and *Flavobacteriaceae*. The first four of these families have the capability of directly utilizing carbohydrates or degrading cellulolytic materials^36-39^. Because the level of organic carbon is 2.3 folds higher in Lake Mendota, our results suggest that the carbon sources in the two lakes do not substantially affect the growing (blooming) period of these families, which otherwise would evoke differences in the growth of their OTUs. In contrast, the six families with the largest differences in peak lengths, confirmed by the smallest *p*-values, include *Nitrosomonadaceae*, *Beijerinckiaceae*, *Cryptosporangiaceae*, *Phyllobacteriaceae*, *Rickettsiaceae*, and *Holophagaceae*. The first three of these families have some capability of using ammonia or fixing nitrogen^19,40-42^, suggesting that the nitrogen availability between the two lakes is different. This observation is partially supported by the differences in the measured concentrations of nitrogen compounds (Table S1). Interestingly, our results suggest that the differences in concentrations of nitrogen compounds between the two lakes might affect the characteristics of the microbial metapopulation, whereas the differences in organic carbon concentration do not.


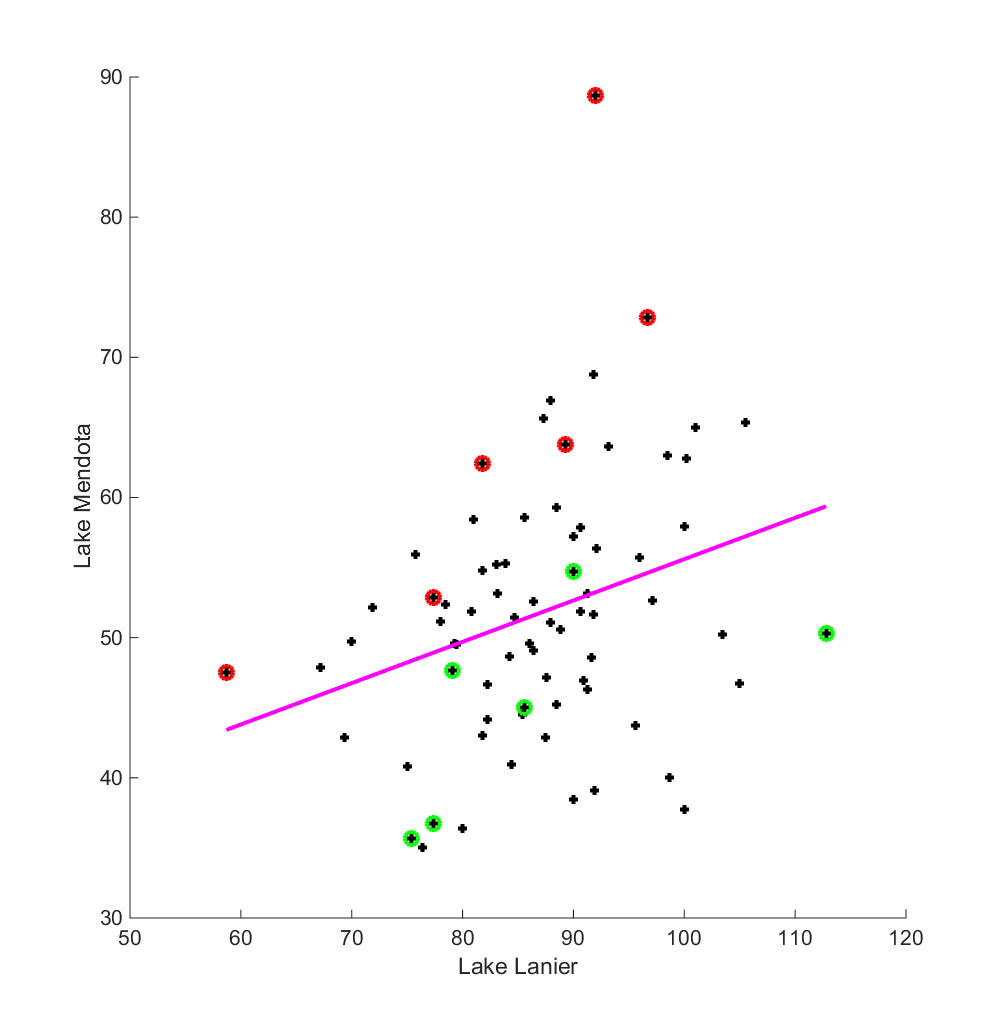


**Figure S10.** **Comparison of blooming periods between Lake Lanier and Lake Mendota for same taxa.** OTUs were grouped as the family level and the blooming period of the same family was compared in the Lanier vs. Mendota data. The x-axis represents the inferred length of each OTU blooming period (in days) for Lake Lanier, while the y-axis shows the corresponding value for Lake Mendota. Clearly, the blooming periods in Lake Mendota tend to be much shorter than for Lake Lanier. The magenta line shows the linear regression trend. The six families with smallest *p*-values or largest *p*-values, according to a *t*-test, are indicated with green and red dots, respectively.

**Supplemental Tables**

**Table S1. Average chemical and physical measurements in Lakes Lanier and Mendota.** Averages were computed monthly from available data. Water temperature is given in ^o^C; concentrations are in mg/L. Averages were computed for months with at least 2 available measurements.

**Table S2. Predicted effects of environmental perturbations on SC abundances.** For each environmental condition (column 1), a perturbation was initiated at the beginning of each month that corresponded to the largest observed difference from the average pattern. Columns 2 and 3 show the mean and standard deviation of these maximum values. Similarly, we computed the number of days that the perturbed and the original predictions are different. These values are displayed in column 4 with standard deviation in column 5. The result shows that the water temperature induced the relatively strongest and longest lasting disturbance on the SCs.

| **Condition** | **Mean of maximal change in response (%)** | **Standard Deviation** | **Mean of median length of response period (days)** | **Standard Deviation** |
| --- | --- | --- | --- | --- |
| **Water Temperature** | 13.73 | 6.45 | 198.52 | 143.5 |
| **pH** | 5.96 | 4.95 | 22.89 | 58.03 |
| **Sulfate** | 1.65 | 2.49 | 12.32 | 48.79 |

**Table S3. Estimated *α_ii_* and *β_i_*_1_ values.**  Means (columns 2 and 4) and standard deviations (columns 3 and 5) of *α_ii_* and *β_i_*_1_ parameters estimated from the dynamic model. The model reported here includes one environmental condition (water temperature), and statistics were obtained from the 193 best-performing model instantiations.

**
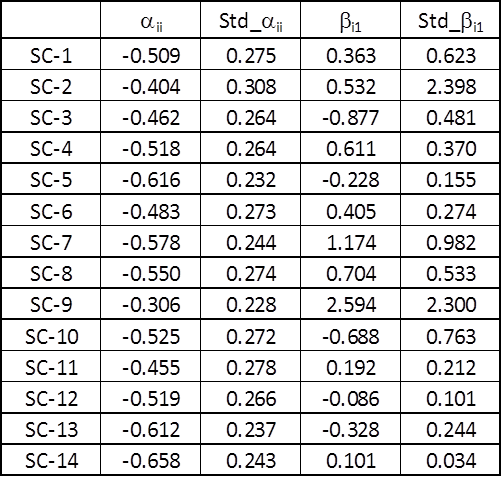
**

**Table S4a. Predicted pairwise co-abundances of mapped OTUs.** Only OTUs with significant co-abundances in small clusters are shown. The table includes cluster ID (1), assigned taxonomy of the genomes (2) and Silva ID (3).

**Table S4b. Predicted pairwise co-abundances between assembled genomes.** Only significant co-abundances are shown. The table includes the genomes, identified by ID number (column 1, 2), resulting *p*-value and slope of the regression line (3, 4) and the assigned taxonomy of the genomes (5,6).

**Table S5. Means of estimated *α_ij_* values.**  Means of *α_ij_* values estimated from the dynamic model. The model reported here includes one environmental condition (water temperature), and the statistics pertain to the 193 best-performing model parameterizations.

**Table S5. Standard deviations of estimated *α_ij_* values.**  Standard deviations of the *α_ij_* estimated from the dynamic model. The model reported here includes one environmental condition (water temperature), and the statistics pertain to the 193 best-performing model parameterizations.

**Table S7. ANOVA test results for comparisons of predicted blooming periods between taxonomic groups of OTUs.** The microbes were divided into taxonomical groups (column 1), and the numbers of groups were recorded (column 2). ANOVA was used to test if the blooming periods of OTUs in the groups (at the same taxonomical level) were significantly different; the associated *p*-value is shown in column 3. Column 4 shows the number of pairwise tests with significant (Bonferroni corrected) *p*-values at each taxonomical level. The Bonferroni-corrected *p*-values suggest a significant difference between the lengths of the blooming periods between groups only at the domain, phylum and class levels. At the domain level, the blooming periods of bacteria and microscopic eukaryotes differ significantly.


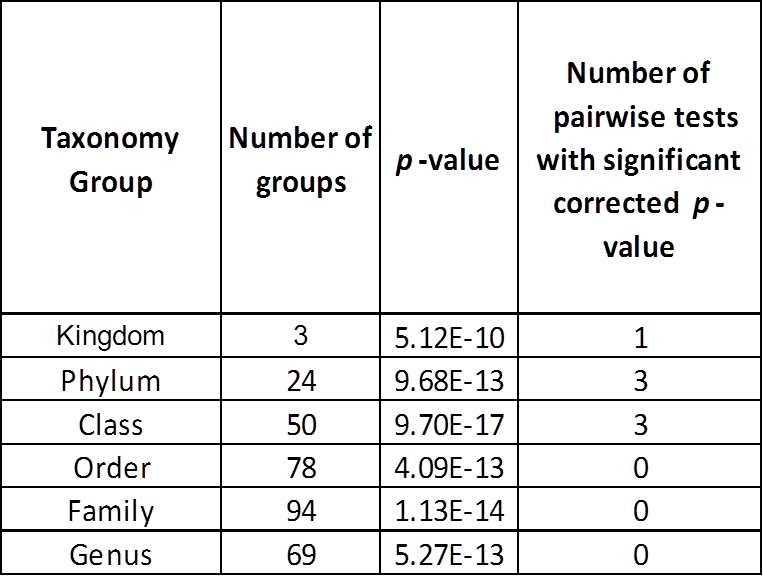


**References**

1 Quast, C. *et al.* The SILVA ribosomal RNA gene database project: improved data processing and web-based tools. *Nucleic acids research* **41**, D590-596, doi:10.1093/nar/gks1219 (2013).

2 Pruesse, E. *et al.* SILVA: a comprehensive online resource for quality checked and aligned ribosomal RNA sequence data compatible with ARB. *Nucleic acids research* **35**, 7188-7196 (2007).

3 Oh, S. *et al.* Metagenomic insights into the evolution, function, and complexity of the planktonic microbial community of Lake Lanier, a temperate freshwater ecosystem. *Applied and environmental microbiology* **77**, 6000-6011, doi:10.1128/AEM.00107-11 (2011).

4 Environmental Protection Division (Georgia Department of Natural Resources). Water Quality Data. (2016).

5 Dam, P., Fonseca, L. L., Konstantinidis, K. T. & Voit, E. O. Dynamic models of the complex microbial metapopulation of lake mendota. *Nature pj Systems Biology And Applications* **2**, 16007 (2016).

6 NTL-LTER. *11 year time series*, <<http://lter.limnology.wisc.edu>> (2011).

7 NTL-LTER. *Chemical Limnology of North Temperate Lakes LTER Primary Study Lakes: Nutrients, pH and Carbon*, <<http://lter.limnology.wisc.edu>> (2013).

8 Neter, J., Wasserman, W. & Kutner, M. H. *Applied Linear Statistical Models Regression, Analysis of Variance, and Experimental Designs*. Third edn, 443-452 (Richard D. Irwin, Inc., 1990).

9 Voit, E. O., Martens, H. A. & Omholt, S. W. 150 years of the mass action law. *PLoS Comput Biol* **11**, e1004012, doi:10.1371/journal.pcbi.1004012 (2015).

10 Ives, A. R. Predicting the response of populations to environmental change. *Ecology* **76**, 926-941 (1995).

11 Hampton, S. E. *et al.* Quantifying effects of abiotic and biotic drivers on community dynamics with multivariate autoregressive (MAR) models. *Ecology* **94**, 2663-2669 (2013).

12 Certain, G., Barraquand, F. & Gårdmark, A. How do MAR(1) models cope with hidden nonlinearities in ecological dynamics? *Methods Ecol. Evol.* **9**, 1975-1995 (2018).

13 Chou, I. C. & Voit, E. O. Recent developments in parameter estimation and structure identification of biochemical and genomic systems. *Mathematical biosciences* **219**, 57-83, doi:DOI 10.1016/j.mbs.2009.03.002 (2009).

14 Voit, E. O. & Chou, I.-C. Parameter estimation in canonical biological systems models. *Int. J. Syst. Synth. Biol.* **1**, 1-19 (2010).

15 Voit, E. O. & Almeida, J. Decoupling dynamical systems for pathway identification from metabolic profiles. *Bioinformatics* **20**, 1670-1681 (2004).

16 Voit, E. O. & Savageau, M. A. Power-law approach to modeling biological systems; III. Methods of analysis. *J Ferment Technol* **60**, 223-241 (1982).

17 Varah, J. M. A spline least squares method for numerical parameter estimation in differential equations. *SIAM J. Sci. Stat. Comput.* **3**, 28-46 (1982).

18 Chou, I.-C. & Voit, E. O. Recent developments in parameter estimation and structure identification of biochemical and genomic systems. *Mathematical biosciences* **219**, 57-83 (2009).

19 Prosser, J., Head, I. & Stein, L. in *The Prokaryotes* (ed Eugene Rosenberg et al.) 901-918 (Springer, 2014).

20 Gallego, V., Sanchez-Porro, C., Garcia, M. T. & Ventosa, A. *Roseomonas aquatica sp nov*., isolated from drinking water. *Int J Syst Evol Micr* **56**, 2291-2295 (2006).

21 Rihs, J. D. *et al.* Roseomonas, a new genus associated with bacteremia and other human infections. *J Clin Microbiol* **31**, 3275-3283 (1993).

22 Guo, M. *et al.* Genome sequences of three species in the family *Planctomycetaceae*. *J. Bacter.* **194**, 3740-3741 (2012).

23 Sohn, J. H., Kwon, K. K., Kang, J. H., Jung, H. B. & Kim, S. J. *Novosphingobium pentaromativorans sp. nov.*, a high-molecular-mass polycyclic aromatic hydrocarbon-degrading bacterium isolated from estuarine sediment. *International journal of systematic and evolutionary microbiology* **54**, 1483-1487 (2004).

24 Ryan, M. P. & Adley, C. C. The antibiotic susceptibility of water-based bacteria Ralstonia pickettii and Ralstonia insidiosa. *Journal of medical microbiology* **62**, 1025-1031, doi:10.1099/jmm.0.054759-0 (2013).

25 Hahn, M. W. *et al.* Polynucleobacter cosmopolitanus sp. nov., free-living planktonic bacteria inhabiting freshwater lakes and rivers. *International journal of systematic and evolutionary microbiology* **60**, 166-173, doi:doi:10.1099/ijs.0.010595-0 (2010).

26 Oren, A. in *The Prokaryotes: Alphaproteobacteria and Betaproteobacteria* (eds Eugene Rosenberg *et al.*) 975-998 (Springer Berlin Heidelberg, 2014).

27 Eder, W. *et al.* Description of Undibacterium oligocarboniphilum sp. nov., isolated from purified water, and Undibacterium pigrum strain CCUG 49012 as the type strain of Undibacterium parvum sp. nov., and emended descriptions of the genus Undibacterium and the species Undibacterium pigrum. *International journal of systematic and evolutionary microbiology* **61**, 384-391, doi:doi:10.1099/ijs.0.018648-0 (2011).

28 Im, W.-T. *et al.* Variovorax ginsengisoli sp. nov., a denitrifying bacterium isolated from soil of a ginseng field. *International journal of systematic and evolutionary microbiology* **60**, 1565-1569, doi:doi:10.1099/ijs.0.014514-0 (2010).

29 Liu, L. *et al.* High correlation between genotypes and phenotypes of environmental bacteria Comamonas testosteroni strains. *BMC Genomics* **16**, 110, doi:10.1186/s12864-015-1314-x (2015).

30 Yu, Z., Yang, J. & Liu, L. Denitrifier Community in the Oxygen Minimum Zone of a Subtropical Deep Reservoir. *PloS one* **9**, e92055, doi:10.1371/journal.pone.0092055 (2014).

31 Morris, R. M. *et al.* SAR11 clade dominates ocean surface bacterioplankton communities. *Nature* **420**, 806-810, doi:10.1038/nature01240 (2002).

32 Tripp, H. J. *et al.* SAR11 marine bacteria require exogenous reduced sulphur for growth. *Nature* **452**, 741-744, doi:10.1038/nature06776 (2008).

33 Jiang, C. Y., Dai, X., Wang, B. J., Zhou, Y. G. & Liu, S. J. Roseomonas lacus sp. nov., isolated from freshwater lake sediment. *International journal of systematic and evolutionary microbiology* **56**, 25-28, doi:10.1099/ijs.0.63938-0 (2006).

34 Rodrigues, J. L. M. & Isanapong, J. in *The Prokaryotes: Other Major Lineages of Bacteria and The Archaea* (eds Eugene Rosenberg *et al.*) 751-756 (Springer Berlin Heidelberg, 2014).

35 Balkwill, D. L., Fredrickson, J. K. & Romine, M. F. in *The Prokaryotes: Volume 7: Proteobacteria: Delta, Epsilon Subclass* (eds Martin Dworkin *et al.*) 605-629 (Springer New York, 2006).

36 Chassard, C., Delmas, E., Robert, C., Lawson, P. A. & Bernalier-Donadille, A. Ruminococcus champanellensis sp. nov., a cellulose-degrading bacterium from human gut microbiota. *International journal of systematic and evolutionary microbiology* **62**, 138-143, doi:10.1099/ijs.0.027375-0 (2012).

37 Janssen, P. H. & Hedlund, B. P. in *Bergey’s Manual of Systematic Bacteriology* Vol. 4 (eds N.R. Krieg *et al.*) 837 (Springer Verlag, 2011).

38 Rosenberg, E. in *The Prokaryotes: Other Major Lineages of Bacteria and The Archaea* (eds Eugene Rosenberg *et al.*) 493-495 (Springer Berlin Heidelberg, 2014).

39 Stackebrandt, E. in *The Prokaryotes: Actinobacteria* (eds Eugene Rosenberg *et al.*) 5-12 (Springer Berlin Heidelberg, 2014).

40 Bernardet, J. F. & Bowman, J. P. in *The Prokaryotes: Actinobacteria* (eds Eugene Rosenberg *et al.*) 481-531 (Springer Berlin Heidelberg, 2014).

41 Marín, I. & Arahal, D. R. in *The Prokaryotes: Alphaproteobacteria and Betaproteobacteria* (eds Eugene Rosenberg *et al.*) 115-133 (Springer Berlin Heidelberg, 2014).

42 Willems, A. in *The Prokaryotes: Alphaproteobacteria and Betaproteobacteria* (eds Eugene Rosenberg *et al.*) 355-418 (Springer Berlin Heidelberg, 2014).
